# Molecular oxygen-enabled photocatalyzed oxygenative coupling *via* C–H functionalization: advances and mechanistic insights

**DOI:** 10.1039/d6ra06747f

**Published:** 2026-09-15

**Authors:** Ambuj Kumar Kushwaha, Pooja Kumari, Sundaram Singh

**Affiliations:** a Department of Chemistry, Indian Institute of Technology (BHU) Varanasi-221005 U.P. India sundaram.apc@iitbhu.ac.in +91 9451658650; b Department of Chemistry, Janta Vedic College Baraut Baghpat-250611 U.P. India ambuj.bhu.chemistry@gmail.com

## Abstract

Visible-light photoredox catalysis has emerged as a powerful platform for the activation and functionalization of inherently inert C–H bonds, enabling synthetically valuable transformations under mild, sustainable, and operationally simple conditions. Among the oxidants employed in these processes, molecular oxygen holds particular significance owing to its abundance, low cost, and dual function as both a terminal oxidant and an oxygen atom source. This perspective provides a concise yet comprehensive overview of recent advancements in oxygen-enabled photocatalytic C–H functionalization, with a focus on oxygenative C–C, C–N, C–S and C–O coupling reactions. Developments in transition metal-catalyzed and metal-free photoredox systems are critically discussed. Mechanistic insights into reactive oxygen species generation, and radical intermediacy are highlighted to elucidate the underlying principles governing these transformations. Collectively, the advances summarized herein underscore the growing impact of O_2_ driven photoredox catalysis in delivering efficient, atom-economic, and environmentally benign routes for the construction of diverse molecular architectures, thereby reinforcing its importance in contemporary organic synthesis.

## Abstract 2

This perspective highlights visible-light photoredox-enabled oxygenative C–H functionalization using molecular oxygen as a sustainable oxidant, covering metal-free and transition-metal catalyzed strategies for C–C and C-heteroatom bond formation.

## Graphical abstract

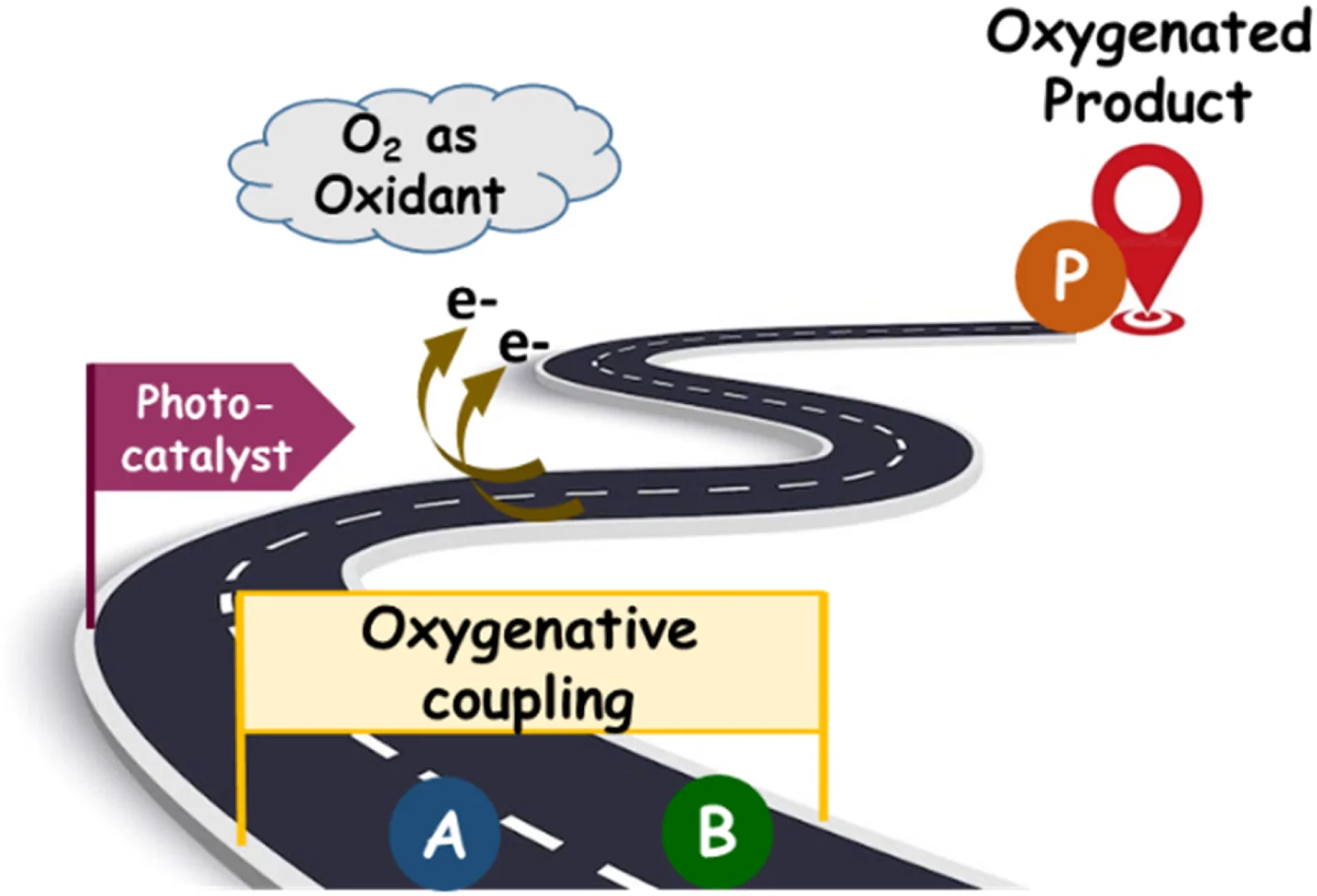

C–H functionalization *i.e.*, activation of inert C–H bonds, which includes a C–H activation step followed by C–H transformation, has emerged as a powerful tool in synthetic organic chemistry.^1–3^ This has been used in a variety of contexts, such as the late-stage functionalization of pharmaceutical derivatives,^4,5^ petroleum feedstock,^6^ polymers,^7^ and the selective functionalization of natural materials.^8,9^ Catalytic C–H functionalization tactics have also been adopted in industry, particularly in agrochemical discovery and pharmaceutical settings.^4^ In the early years of this century, the inert C–H bond functionalization reaction seemed very difficult to achieve. Low reactivity,^10^ greater bond dissociation energies (BDE),^11^ and the non-polar nature^12^ of the isolated C–H bonds are some of the major obstacles to C–H bond activation. The low acidity (p*K*_a_ = 45–60) and high bond energies (usually 90–100 kcal mol^−1^) of C–H bonds are frequently blamed for their inadequate reactivity.^13^

Furthermore, the distinction between “inert” and “reactive” may become hazier due to the recent resurgence of photocatalysis, since an inert C–H bond in its ground state might become very active when activated through photo-catalyzed energy/electron transfer.^14–21^ With its potential to transform visible light into chemical energy, photocatalysis fully embraces the culture of green chemistry.^21–27^ In organic synthesis, photoredox catalysis is a sustainable and efficient option that permits the gentle production of difficult carbon–heteroatom bonds.^28–33^ There have been numerous reviews and perspective articles on the subject of photoredox catalysis in general.^21,34–40^ This perspective aims to provide a comprehensive overview of the application of photoredox catalysis in C–H functionalization transformations reported during the period from 2015 to 2026.

## 1 Oxygen as an oxidant and an oxygen atom source

Merging the concept of C–H functionalization and photocatalysis for oxygenative coupling reveals a green, effective alternative for selective carbon–heteroatom and carbon–carbon cross-coupling reactions^41–45^ under mild conditions (Fig. 1). Naturally, the synthesis of O-containing compounds with molecular oxygen as both an oxidant and an oxygen atom source is especially attractive. This kind of oxygen incorporation into organic substrates with molecular oxygen, also referred to as oxygenation reaction,^46–49^ has made great progress in recent years with excellent atom economy and low environmental impact. In nature, oxygenase can directly incorporate an oxygen atom from molecular oxygen into the target molecule during biological synthesis.^50^ These enzymatic processes have inspired chemists to search for synthetic analogues, as oxygen (or even better air) is cheaper, less polluting, and produces mostly water as a by-product.

**Fig. 1:**
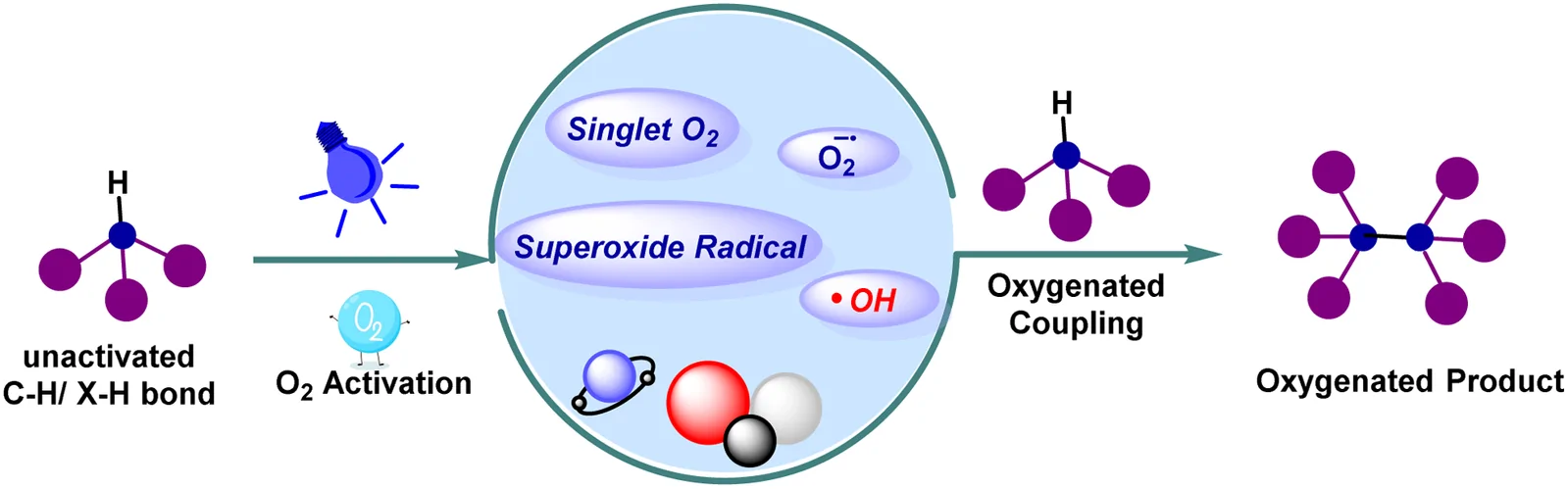
Photoinduced oxygenative coupling using O_2_ as an oxidant.

However, in order to be used as an oxidant, O_2_ must be activated into a reactive oxygen species (ROS), such as singlet oxygen, superoxide anion radical, hydroxyl radical, or hydrogen peroxide, due to the spin-forbidden reaction.^51–54^ One of the most promising and effective methods for molecular oxygen activation in order to carry out sustained oxygenation activities is visible light photocatalysis.^55–57^ From an environmentally friendly and sustainable perspective, the best technique for creating molecules containing oxygen is to directly use O_2_ as an oxidant under visible light irradiation.^49,58–60^

Oxygen-containing molecules are ubiquitous in natural products and bioactive compounds; they are also important synthons in organic synthesis.^61–63^ Indeed, a large number (>200) of oxygen-containing drugs have been marketed worldwide, including widely used antibiotics, anticoagulants, and cholesterol-lowering statins.^64–67^

The term “oxygenation” refers to the incorporation of molecular oxygen into the product in addition to its function as an electron acceptor. As a reagent, there are primarily three ways to add molecular oxygen to the product: singlet oxygen ^1^O_2_, which is typically created by energy transfer between the excited photocatalyst and ^3^O_2_, superoxide radical anion produced by a single electron transfer (SET) process, and triplet ^3^O_2_, which functions as a radical trapping agent (Scheme 1). While several comprehensive reviews have addressed the light-driven photocatalytic activation of molecular oxygen as an oxidant for oxygenation processes, this article specifically emphasizes recent advancements in employing oxygen as an oxygen atom donor for visible-light-mediated oxygenative coupling reactions.^68–70^

**Scheme 1:**
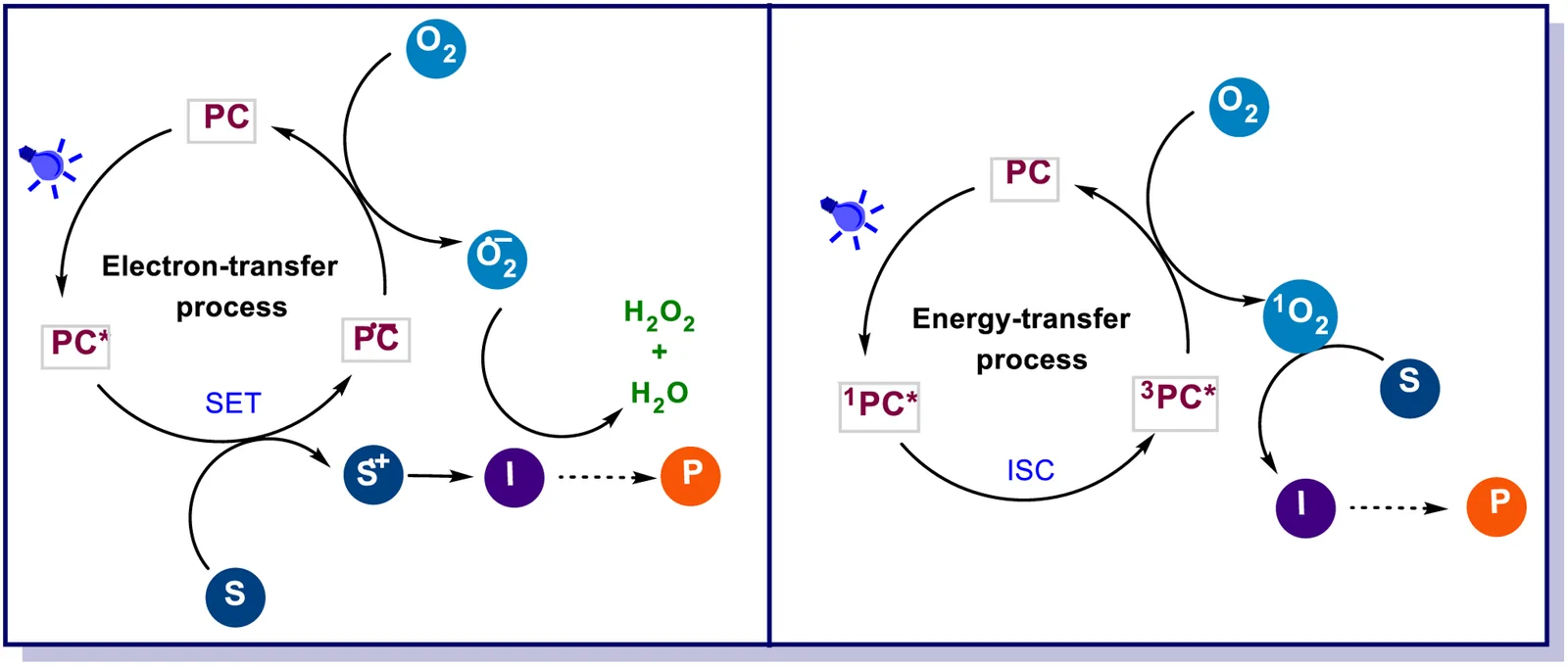
Pathways to incorporate molecular oxygen into the product.

## 2 C–H functionalized oxygenative coupling

At the start of the century, activating C–H bonds, *i.e.*, C–H functionalization, posed a significant challenge due to the low reactivity of these bonds. In today's context, this area has emerged as one of the extensively investigated areas for synthetic chemists. This includes both the breadth of feasible transformations and the variety of catalytic methods facilitating C–H functionalization. The field of photoredox catalysis for C–H bond functionalization is rapidly expanding and intersecting with various scientific disciplines, including renewable energy and chemical feedstock applications, novel reaction discovery, natural product synthesis, materials science, and biological applications.

### 2.1. Photoredox-catalyzed C–C oxygenative coupling reactions

The essence of chemical synthesis lies in building chemical bonds, their remarkable ability to form carbon–carbon bonds, facilitating the efficient synthesis of complex molecules from basic starting materials. Thus, visible-light-initiated C–C coupling has emerged as an indispensable, with carbon–carbon (C–C) bonds serving as crucial structural elements in numerous bioactive compounds, natural products, and functional materials. Therefore, the formation of these bonds is a fundamental process in synthetic organic chemistry. Photoinduced C–C coupling reactions have gained prominence due to economical, and eco-friendly tool in chemical synthesis.^33,71–75^

Chen and co-workers in 2015 developed a mild and metal-free photocatalytic method for hydroperoxyarylation of styrenes using aryl hydrazines and ambient air as the oxidant (Scheme 2a).^76^ This transformation proceeds under visible light (23 W CFL) using Remazol Brilliant Blue R (RBBR) dye as an organic photosensitizer. The method enables the simultaneous formation of C–C and C–O bonds, producing hydroperoxides in moderate to excellent yields. Importantly, the reaction uses simple aryl hydrazines instead of unstable aryldiazonium salts and avoids external oxidants or transition metals. The methodology shows broad compatibility with various styrenes and aryl hydrazines. Electron-donating and electron-withdrawing groups on the styrene ring (*e.g.*, –F, –Cl, –Me, –OMe) at *para*-, *meta*-, and *ortho*-positions were well tolerated. 1,1-Disubstituted styrenes gave higher yields, likely due to the stability of tertiary radicals. Naphthyl-substituted alkenes also worked efficiently. Electron-deficient aryl hydrazines gave lower yields than phenylhydrazine, while alkyl hydrazines were unreactive. Acrylate derivatives like methyl methacrylate underwent successful hydroperoxyarylation, but other electron-poor or sterically hindered alkenes were generally unreactive. Heteroaromatic olefins (*e.g.*, 4-vinylpyridine, vinylthiophene) also failed to react.

**Scheme 2:**
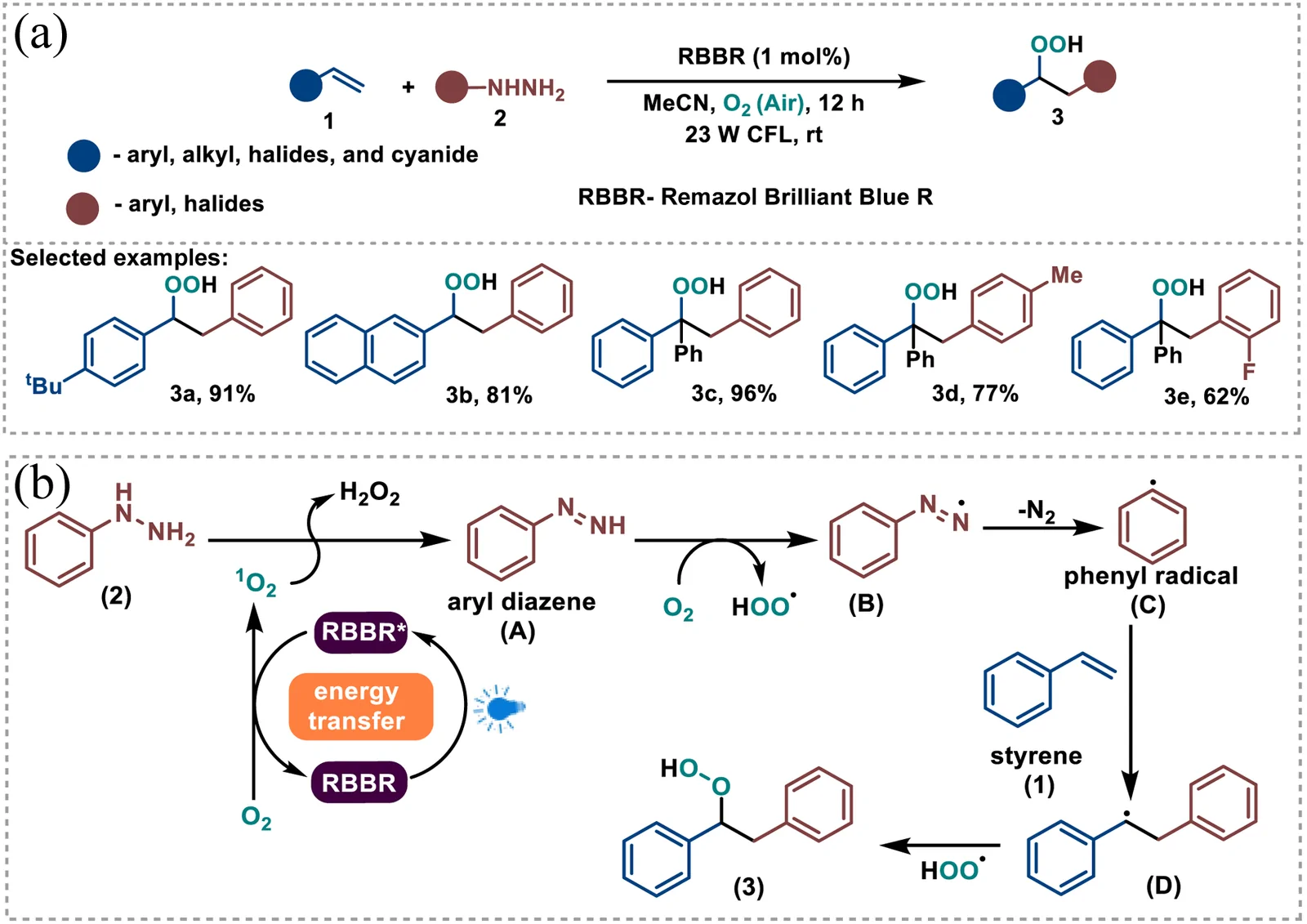
(a) Hydroperoxyarylation of styrenes using aryl hydrazines. (b) Proposed mechanism for hydroperoxyarylation of styrenes using aryl hydrazines.

Mechanistic studies suggest a singlet oxygen-mediated radical pathway (Scheme 2b). Under visible light, RBBR acts as a photosensitizer to generate singlet oxygen, which oxidizes aryl hydrazines to aryl diazenes (A). These diazenes undergo further oxidation to aryl radicals (C). The aryl radicals add to the alkene to form a benzylic radical intermediate (D), which is trapped by a hydroperoxide radical (also derived from singlet oxygen), yielding the hydroperoxide product. TEMPO trapping experiments confirmed the radical nature of the process. The reaction did not proceed in the dark or under nitrogen, underscoring the essential role of light and dioxygen. The structure and regioselectivity of the products were confirmed *via* NOESY NMR and X-ray crystallography. This work provides a rare example of metal-free photocatalytic carbooxygenation of styrenes and offers a valuable green synthetic route to hydroperoxides using inexpensive dyes, atmospheric oxygen, and visible light under mild conditions.

Ding and co-workers in 2017 developed a sustainable, metal-free protocol for the direct synthesis of aryl ketones *via* visible-light-promoted oxidative radical addition of arylhydrazines to alkenes under ambient air conditions (Scheme 3a).^77^ The reaction is catalyzed by methylene blue and proceeds in the presence of DABCO and air as the terminal oxidant, under blue LED irradiation at room temperature. This green method offers a practical and scalable alternative to traditional metal-catalyzed processes, such as the Wacker oxidation, by avoiding the use of precious metals and harsh reagents. The methodology demonstrates broad substrate tolerance. A variety of styrene derivatives bearing electron-donating or electron-withdrawing groups at *para*, *meta*, and *ortho* positions underwent smooth conversion to the corresponding aryl ketones in good to excellent yields (up to 86%). *ortho*-Substitution slightly diminished the yields due to steric hindrance. β-Substituted alkenes such as β-methylstyrene and divinylbenzenes were also compatible. Additionally, several aryl hydrazines with diverse substitution patterns were tolerated, including those with halogen and methyl groups. The protocol also proved effective on gram-scale, indicating its synthetic applicability.

**Scheme 3:**
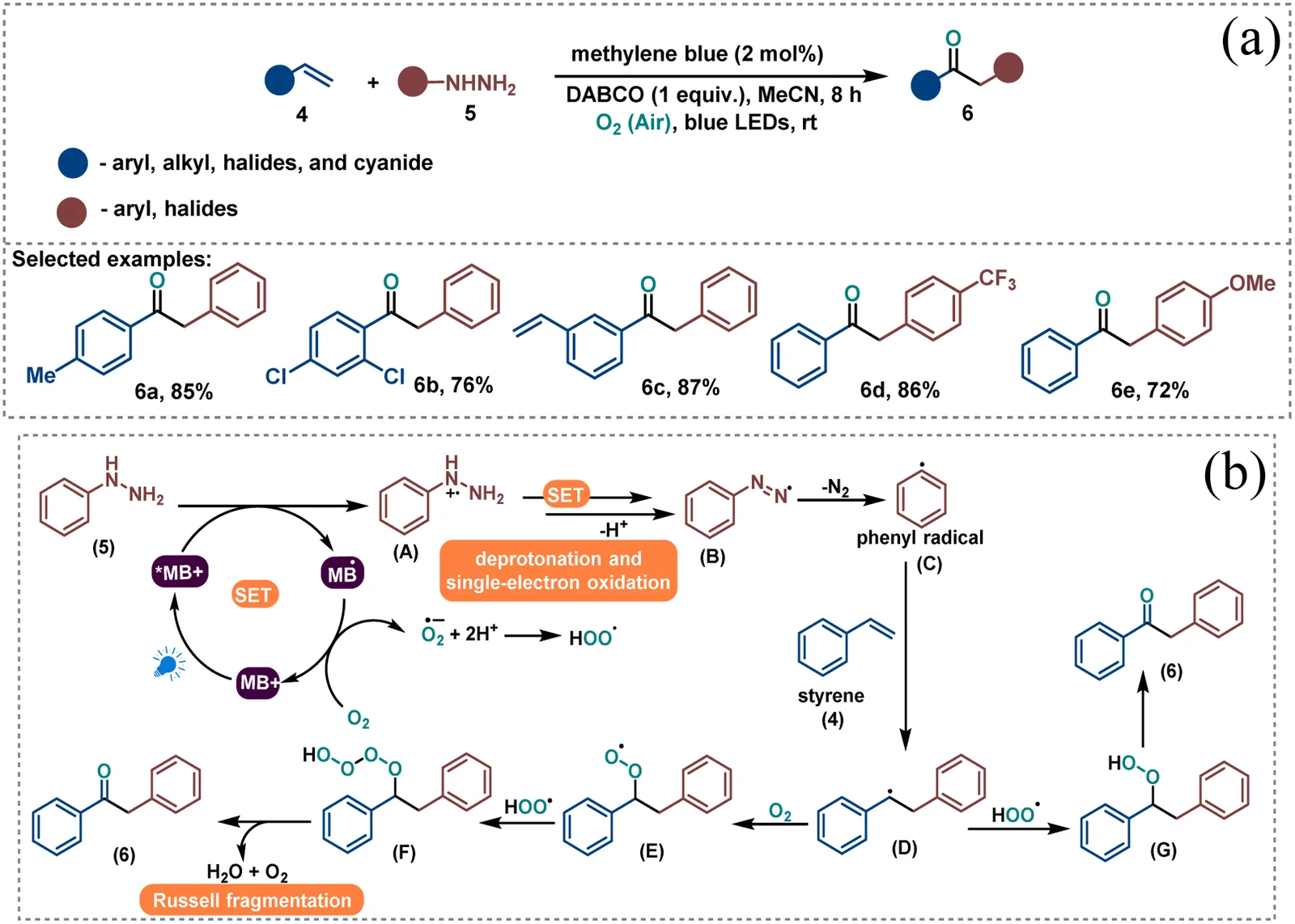
(a) Metal-free synthesis of ketones *via* oxidative radical addition of aryl hydrazines to alkenes. (b) Proposed mechanism for metal-free synthesis of ketones *via* oxidative radical addition of aryl hydrazines to alkenes.

Mechanistic investigations support a radical-mediated pathway initiated by photoexcitation of methylene blue (MB^+^) under blue light (Scheme 3b). The excited-state MB^+^ is reductively quenched by arylhydrazine to form a radical cation (A), which undergoes deprotonation and sequential single-electron oxidation to furnish the aryl radical (C). This radical adds to the alkene to form a carbon-centered radical intermediate (D), which is further oxidized in the presence of molecular oxygen and hydroperoxyl radicals (HOO˙) to generate the corresponding ketone. Two possible oxidative fragmentation pathways-both involving peroxy intermediates and Russell-type rearrangements were proposed to explain the conversion of radical adducts to final ketone products. Stern–Volmer quenching experiments confirmed that arylhydrazines, and not alkenes, are the primary quenchers of the excited photocatalyst. This protocol offers a metal-free, operationally simple, and environmentally benign route to aryl ketones with water and nitrogen as the only byproducts, broadening the utility of arylhydrazines as radical precursors in green synthetic methodologies.

Hwang and co-workers in 2017 developed a visible-light-mediated, copper-catalyzed oxidative coupling of phenols with terminal alkynes, enabling regioselective access to hydroxylated aryl and alkyl ketones *via* C≡C triple bond cleavage under ambient conditions (Scheme 4a).^78^ The transformation employs simple CuCl (5 mol%) and atmospheric oxygen as the oxidant, without any external bases, ligands, or photocatalysts. The reaction tolerates a wide range of phenols and terminal alkynes, providing a modular and sustainable strategy for the synthesis of valuable ketone scaffolds relevant to pharmaceuticals and materials chemistry. The protocol accommodates a broad spectrum of phenols and terminal alkynes, including electron-rich, halogenated, and heteroaryl-substituted substrates. Phenols are readily oxidized *in situ* to benzoquinone intermediates, which subsequently couple with alkynes under light irradiation. The method delivers structurally diverse hydroxylated ketones in moderate to excellent yields. Both aryl and aliphatic alkynes undergo regioselective coupling with high efficiency, with no detectable homocoupling byproducts. The synthetic utility of this transformation was demonstrated in concise, two-step syntheses of pharmaceutically relevant molecules such as pitofenone and fenofibrate, with superior overall yields (72–76%) compared to traditional multistep methods.

**Scheme 4:**
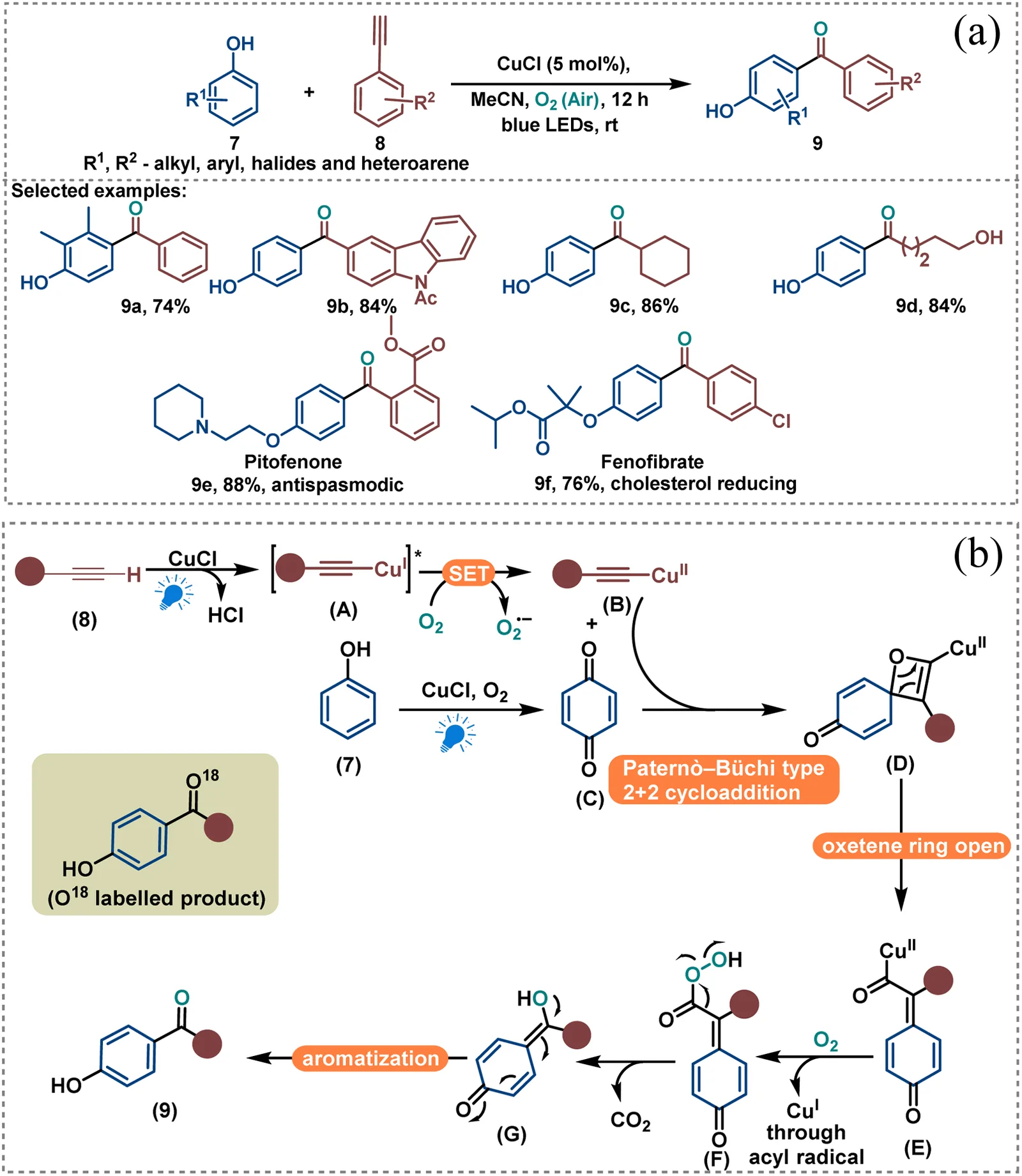
(a) Copper photoredox catalysis for regioselective synthesis of hydroxylated ketones. (b) Proposed mechanism for copper photoredox catalysis for regioselective synthesis of hydroxylated ketones.

Mechanistic studies indicate a photoinduced single-electron transfer (SET) process initiated by *in situ* generated Cu(i)-phenylacetylide species (A) (Scheme 4b). Upon visible light excitation, this complex donates an electron to molecular oxygen, producing a Cu(ii)-acetylide species (B) and a superoxide radical anion. The superoxide facilitates phenol oxidation to benzoquinone (C), which engages in a Paternò–Büchi-type [2 + 2] cycloaddition with the Cu(ii)-acetylide to form a strained oxetene intermediate (D). Subsequent fragmentation and radical rearrangements, including acyl radical generation and CO_2_ extrusion, culminate in the formation of the hydroxylated ketone product. ^18^O_2_-labeling and TEMPO inhibition experiments confirm the involvement of molecular oxygen as the oxygen source and a radical-based pathway.

Sultan and co-workers in 2019 reported a visible-light-promoted protocol for the oxidative coupling of terminal alkynes with 1,4-benzoquinones and 1,4-naphthoquinones using potassium persulfate (K_2_S_2_O_8_) as oxidant and trifluoroacetic acid (TFA) under blue LED irradiation (Scheme 5a).^79^ This methodology operates under mild, metal-free conditions and leverages radical-mediated activation of unactivated alkynes. The method shows excellent functional group tolerance. A variety of aryl- and alkyl-substituted terminal alkynes react with benzoquinones to afford 4-hydroxyaryl and aryl/alkyl ketones in high yields (up to 91%). Substituents such as alkyl, halogen, trifluoromethyl, and methoxy groups were well tolerated. Aliphatic alkynes, including cyclopropyl-, pentyl-, heptyl-, and nonylacetylene, furnished corresponding aryl/alkyl ketones in 81–90% yield.

**Scheme 5:**
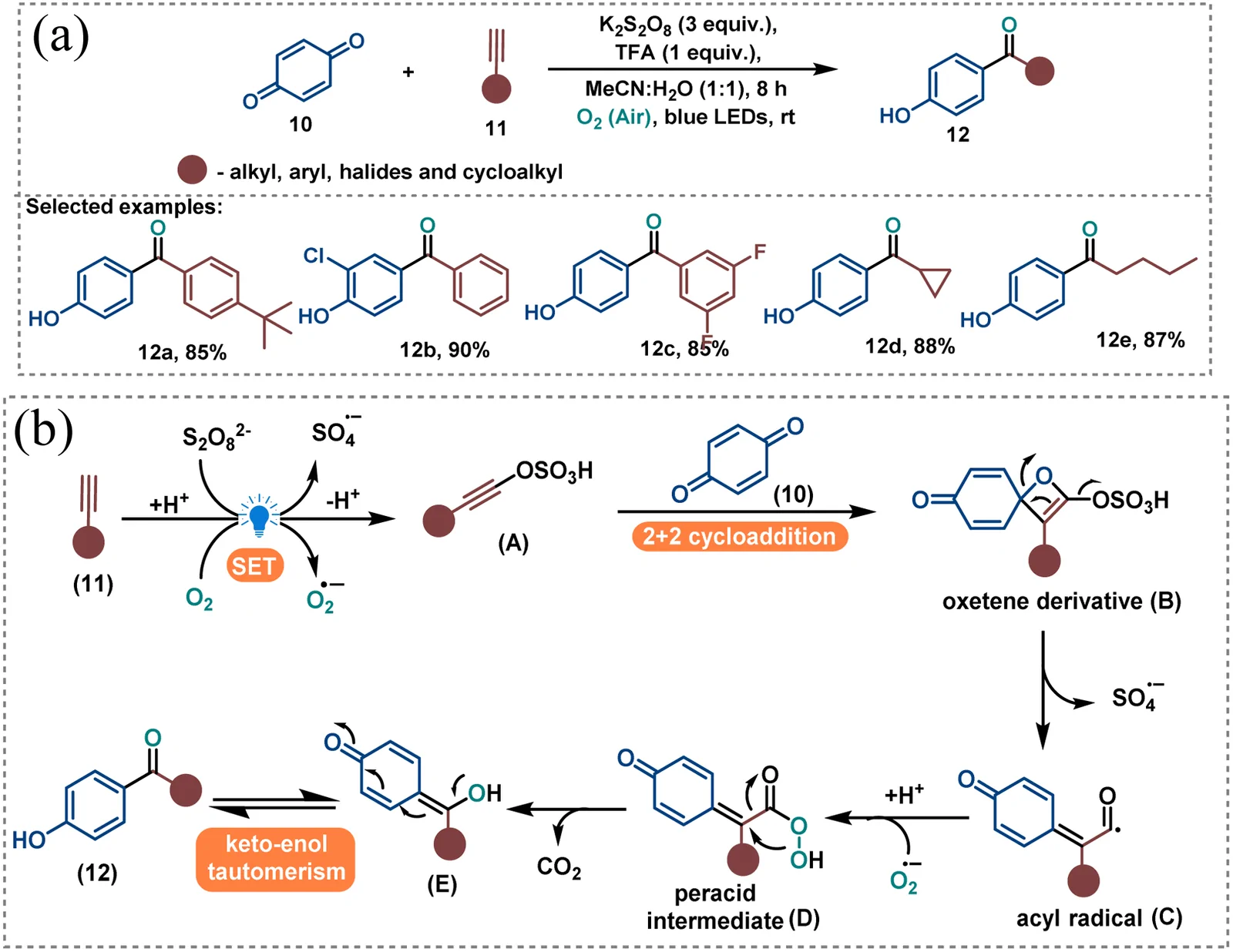
(a) Oxidative coupling of terminal alkynes with 1,4-quinones. (b) Proposed mechanism for oxidative coupling of terminal alkynes with 1,4-quinones.

Mechanistic studies including cyclic voltammetry, radical trapping (*e.g.*, with TEMPO), and dye degradation experiments support a radical-mediated process (Scheme 5b). Photoinduced homolysis of persulfate generates sulfate radical anion (SO_4_˙^−^), which adds to the terminal alkyne to form a vinyl radical. This intermediate undergoes oxygenation to afford a peroxy species, which proceeds through a Paternò–Büchi-type cycloaddition with benzoquinone to generate oxetene intermediates (B) (leading to ketones).

Hajra and co-workers in 2020 developed a metal-free, visible-light-promoted oxidative coupling of vinylarenes (*e.g.*, styrene derivatives) with cyclic ethers to furnish α-oxyalkylated ketones under mild conditions (Scheme 6a).^80^ The reaction utilizes rose bengal as an organic photosensitizer, *tert*-butyl hydrogen peroxide (TBHP) as the oxidant, and proceeds under ambient air at room temperature. The method represents a straightforward, eco-friendly, and efficient approach to generate C–C and C–O bonds simultaneously, affording structurally diverse ketones in moderate to excellent yields. The reaction accommodates a variety of styrenes bearing electron-donating (*e.g.*, –Me, –^*t*^Bu, –OMe, –O^*t*^Bu) and electron-withdrawing (*e.g.*, –F, –Cl, –Br) substituents, delivering the corresponding α-oxyalkylated ketones in good to excellent yields. Extended π-systems such as 4-vinylbiphenyl and 2-vinylnaphthalene, as well as α-methylstyrene, also participated effectively. In addition to tetrahydrofuran, other cyclic ethers including 2-methyltetrahydrofuran, 1,3-dioxolane, tetrahydropyran, and 1,4-dioxane were successfully employed. However, linear alkenes (*e.g.*, 1-octene) and simple ethers like diethyl ether were unreactive. The practicality of the protocol was demonstrated through a gram-scale reaction with no loss in efficiency.

**Scheme 6:**
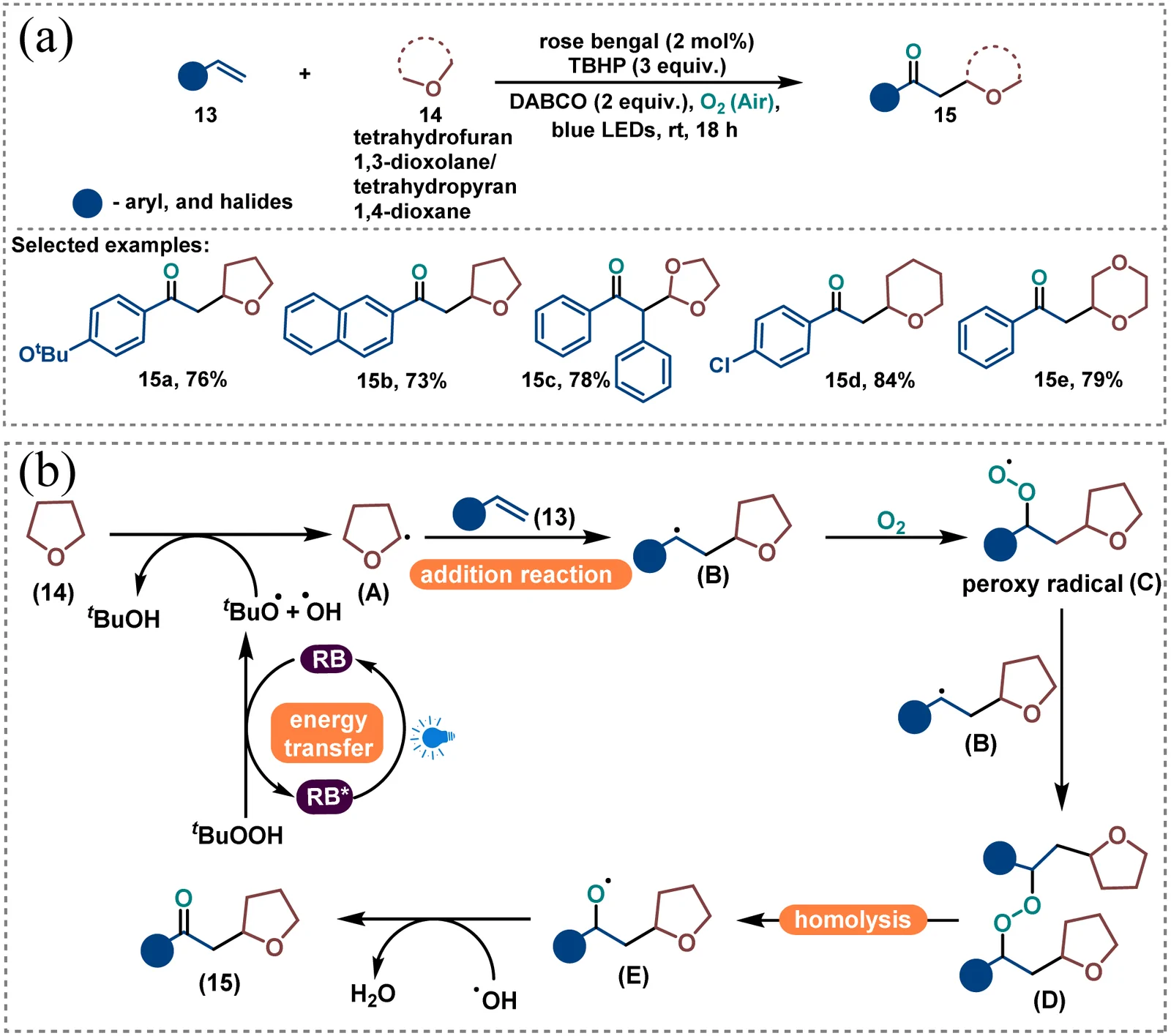
(a) Organophotoredox-mediated oxyalkylation of vinylarenes with cyclic ethers. (b) Proposed mechanism for organophotoredox-mediated oxyalkylation of vinylarenes with cyclic ethers.

Mechanistic studies, including radical trapping experiments with TEMPO and BHT, suggest a radical-based pathway (Scheme 6b). The excited state of rose bengal (RB*) transfers energy to TBHP, generating *tert*-butoxy and hydroxyl radicals. The *tert*-butoxy radical abstracts a hydrogen atom from the cyclic ether, forming an α-oxy radical (A) which adds to the vinylarene to yield a benzylic radical intermediate (B). Subsequent trapping by molecular oxygen and homolytic cleavage of a peroxy intermediate leads to the formation of the ketone product. Control experiments further confirm the requirement of oxygen and the involvement of radical species in the transformation.

Hwang and co-workers in 2020 developed a visible-light-mediated, copper-catalyzed aerobic oxidation of diarylalkynes to α-diketones under mild conditions, utilizing ambient air as the terminal oxidant and copper(ii) chloride (CuCl_2_) as the photocatalyst (Scheme 7a).^81^ This method obviates the need for exogenous photosensitizers or radical initiators and represents a significant advancement in green oxidation protocols. The method accommodates a wide range of diarylalkynes, including those bearing electron-donating, halogen, and electron-withdrawing substituents, affording α-diketones in good to excellent yields. While thiophene-containing alkynes were tolerated, pyridine-based and dialkyl-substituted alkynes showed limited reactivity due to overoxidation.

**Scheme 7:**
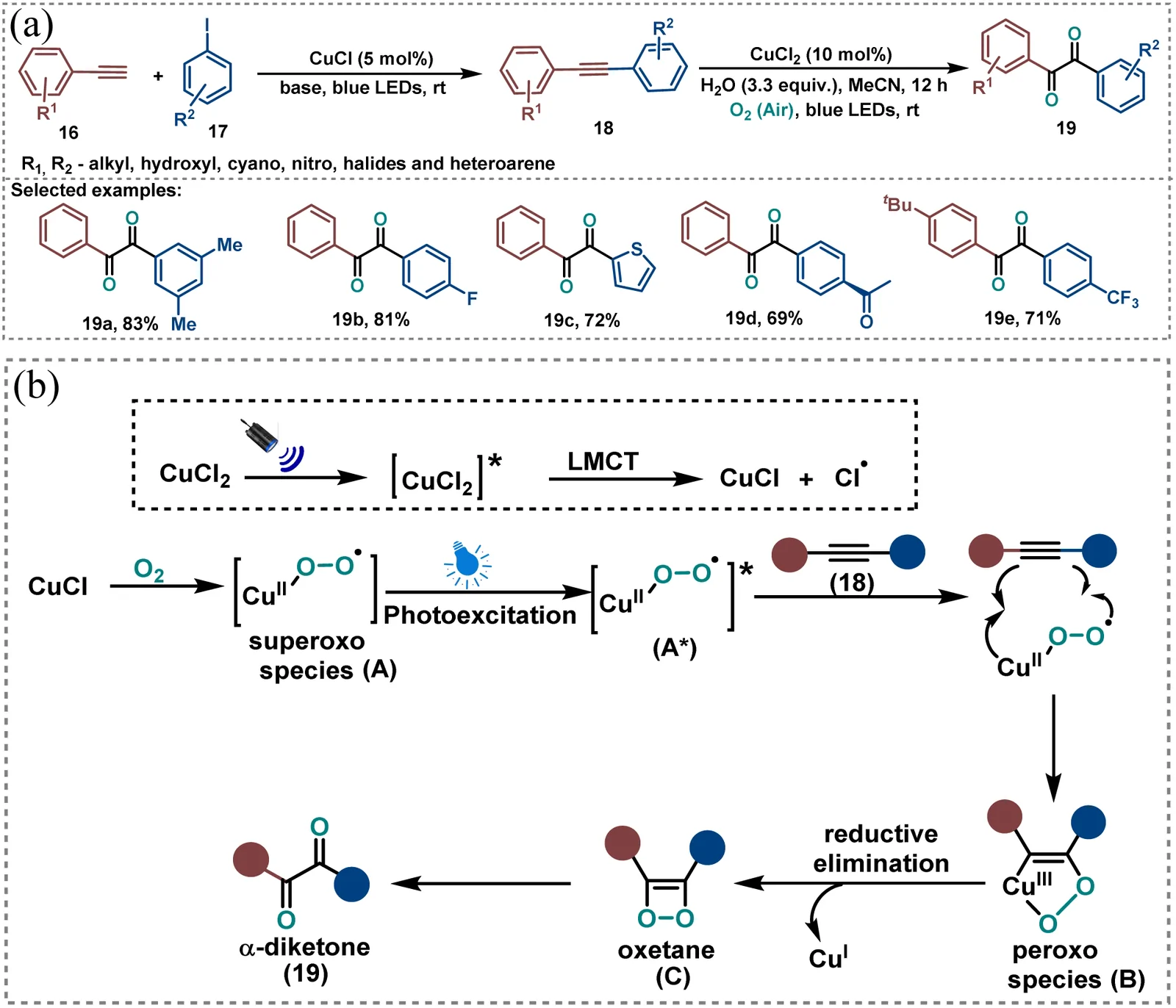
(a) Oxidation of diarylalkynes to α-diketones. (b) Proposed mechanism for oxidation of diarylalkynes to α-diketones.

The proposed mechanism involves photoexcitation of CuCl_2_ to generate Cu(i), which reacts with O_2_ to form a copper(ii)-superoxo species (A) (Scheme 7b). This species, upon further photoactivation, interacts with the diarylalkyne *via* π-coordination, leading to a Cu(iii)-peroxo intermediate (B). Subsequent intramolecular radical cyclization and reductive elimination furnish a 1,2-dioxetane intermediate (C), which decomposes to form the final α-diketone product. The role of water was found to be beneficial in promoting O–O bond cleavage in the intermediate, improving yields. EPR and UV-vis data support the involvement of superoxide and light-absorbing copper intermediates in the reaction pathway.

Hwang and co-workers in 2021 developed a photo-induced, copper-catalyzed aerobic (Csp^2^)–H annulation of amidines with terminal alkynes to access 2,4-disubstituted quinazolines under ambient conditions (Scheme 8a).^82^ This protocol employs inexpensive CuCl and atmospheric oxygen as the catalyst and terminal oxidant, respectively, avoiding external photosensitizers, ligands, or stoichiometric oxidants. The annulation strategy accommodates a broad range of *N*-arylbenzimidamides and terminal aryl or heteroaryl alkynes bearing electron-donating, electron-withdrawing, or halogen substituents, affording the desired quinazoline derivatives in moderate to good yields. Selectivity was preserved in unsymmetrical systems, and the methodology exhibited some tolerance toward aliphatic alkynes, albeit with reduced efficiency. Notably, the reaction was successfully applied to the rapid synthesis of known bioactive quinazoline scaffolds, including anti-cancer compounds, in only three steps from commercially available precursors representing a significant improvement over previously reported six-step thermal syntheses.

**Scheme 8:**
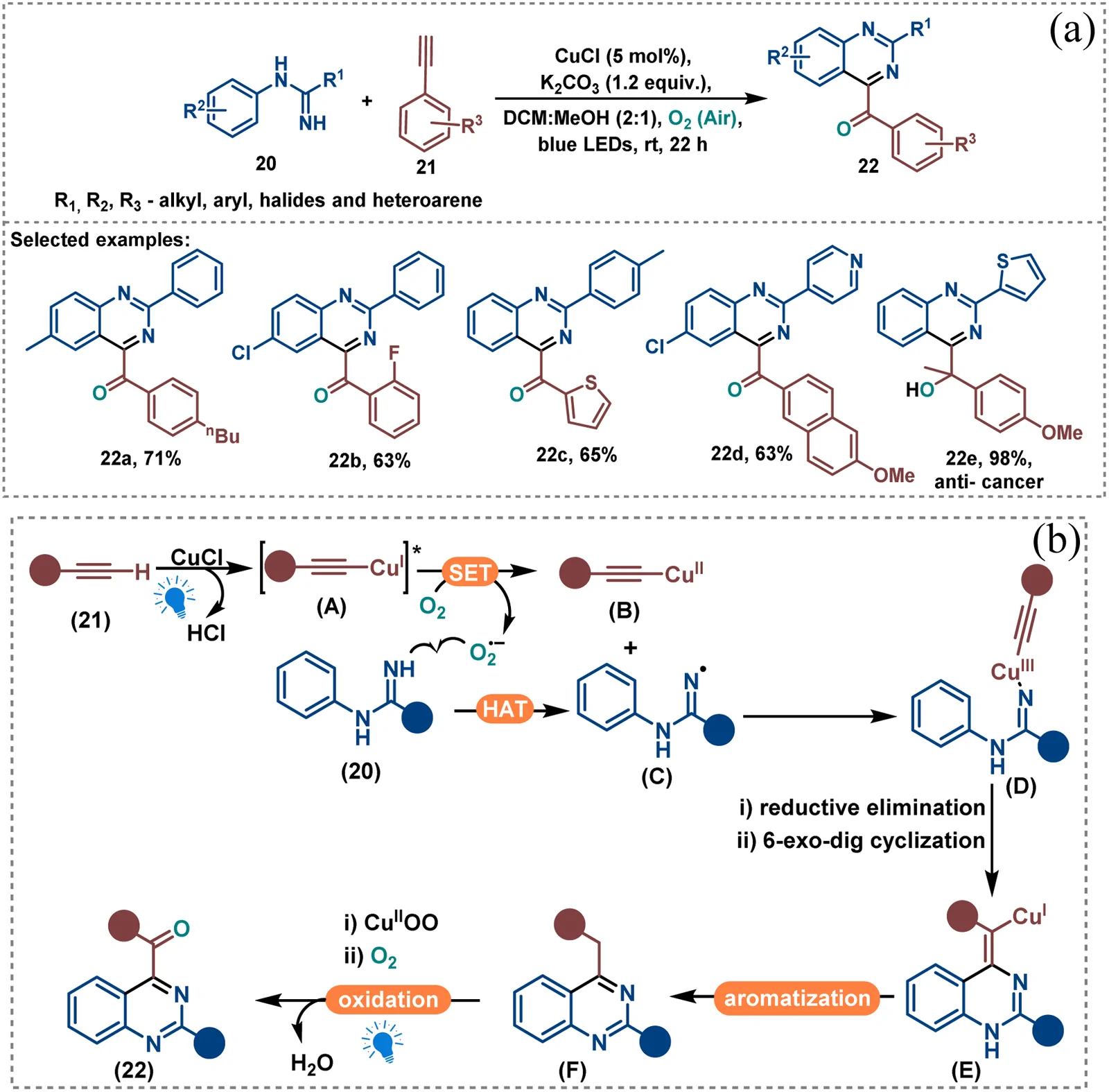
(a) Synthesis of quinazolines *via* aerobic oxidative (Csp^2^)–H annulation of amidines with terminal alkynes. (b) Proposed mechanism for synthesis of quinazolines *via* aerobic oxidative (Csp^2^)–H annulation of amidines with terminal alkynes.

Mechanistic studies, including radical inhibition by TEMPO and ^18^O_2_-labeling experiments, and H_2_O^18^-labeling, support a photo-oxidative pathway initiated by *in situ* generated Cu(i)-phenylacetylide species (A) (Scheme 8b). Upon visible light irradiation, the Cu(i)-acetylide undergoes photoexcitation, followed by single-electron transfer to O_2_, forming a Cu(ii) complex and superoxide radical. This activates the amidine *via* hydrogen abstraction, yielding a nitrogen-centered radical (C) that combines with the Cu(ii) species to form a Cu(iii) intermediate (D). Reductive elimination and subsequent 6-*exo*-dig cyclization afford the quinazoline scaffold, with water as the only byproduct.

Kushwaha and co-workers in 2022 developed an efficient, metal-free, and environmentally benign method for synthesizing 3-methyleneindolin-2-ones *via* visible-light-induced oxidative coupling of indoles with active methylene compounds (Scheme 9a).^83^ Utilizing eosin Y as an organic photocatalyst, and air and water as terminal oxidants, the reaction proceeds under mild conditions at room temperature using blue LED irradiation in a DMF : H_2_O (4 : 1) solvent system. This sustainable approach avoids metal catalysts and generates high yields with no side products. The methodology exhibited a broad scope across various substituted indoles and active methylene partners. Electron-donating and electron-withdrawing groups (*e.g.*, –Me, –Br, –Cl, –F, –NO_2_) on the indole ring were well tolerated. A wide range of active methylene compounds, including malononitrile, ethyl cyanoacetate, dimedone, and barbituric acid, reacted efficiently. Notably, one equivalent of indole reacted with one equivalent of malononitrile, ethyl acetoacetate, or dimedone, while two equivalents of barbituric acid were required, indicating distinct reactivity. The reaction gave excellent isolated yields (up to 88%) for a wide variety of products.

**Scheme 9:**
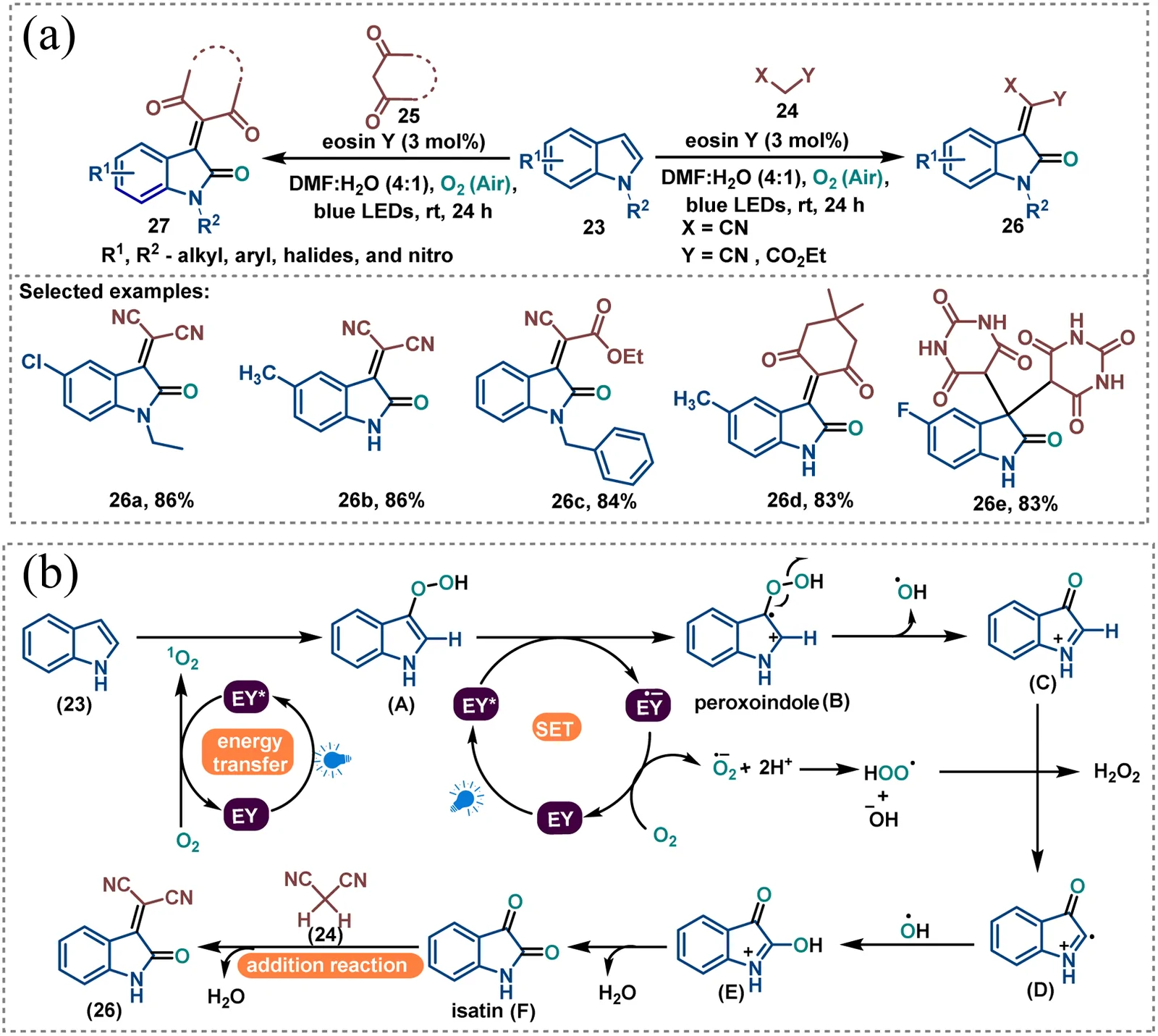
(a) Oxidative coupling of indoles with active methylene compounds. (b) Proposed mechanism for oxidative coupling of indoles with active methylene compounds.

Mechanistic studies and control experiments support a visible-light-induced radical mechanism (Scheme 9b). Upon blue light exposure, eosin Y is excited to its singlet state (EY*), which is quenched by oxygen to generate singlet oxygen and eventually a superoxide radical anion. These reactive oxygen species initiate oxidative transformations of the indole to isatin intermediates (F). Further coupling with active methylene compounds and subsequent dehydration delivers the methylene-oxindole products. Radical trapping experiments (with TEMPO, BHT) significantly suppressed the reaction, and detection of H_2_O_2_ and intermediate isatin confirms the involvement of radical pathways and oxygen as an essential oxidant. This strategy provides a sustainable, scalable, and metal-free route to biologically significant oxindole derivatives, aligning well with the principles of green chemistry and expanding the scope of visible-light photoredox catalysis in C–C bond formation.

Singh and co-workers in 2023 reported a dual catalytic method employing visible light, eosin Y, and Co(OAc)_2_·4H_2_O for the synthesis of unsymmetrical ketones *via* the decarboxylative oxidative coupling of α,β-unsaturated carboxylic acids with aryldiazonium salts (Scheme 10a).^84^ This transition-metal-assisted, photoredox-promoted method proceeds under mild conditions and enables C=C bond cleavage along with C–C and C–O bond formation. The strategy leverages sustainable reaction conditions (blue LED, ambient temperature, air as oxidant) and utilizes inexpensive, readily available starting materials. A broad range of cinnamic acid derivatives and aryldiazonium salts were compatible with the reaction conditions. Electron-rich and electron-deficient diazonium salts, as well as α,β-unsaturated acids bearing aromatic, heteroaromatic, and aliphatic backbones, were successfully employed, affording diaryl and alkyl–aryl ketones in moderate to excellent yields (55–88%). Electron-donating substituents on the aryl ring enhanced yields, while *ortho*-substitution and strong electron-withdrawing groups (*e.g.*, –NO_2_, –CF_3_) slightly diminished reactivity. Aliphatic acids such as crotonic and pentenoic acids also participated, giving the corresponding ketones in up to 77% yield. However, pyridyl and furyl-substituted alkenes were incompatible under the optimized conditions.

**Scheme 10:**
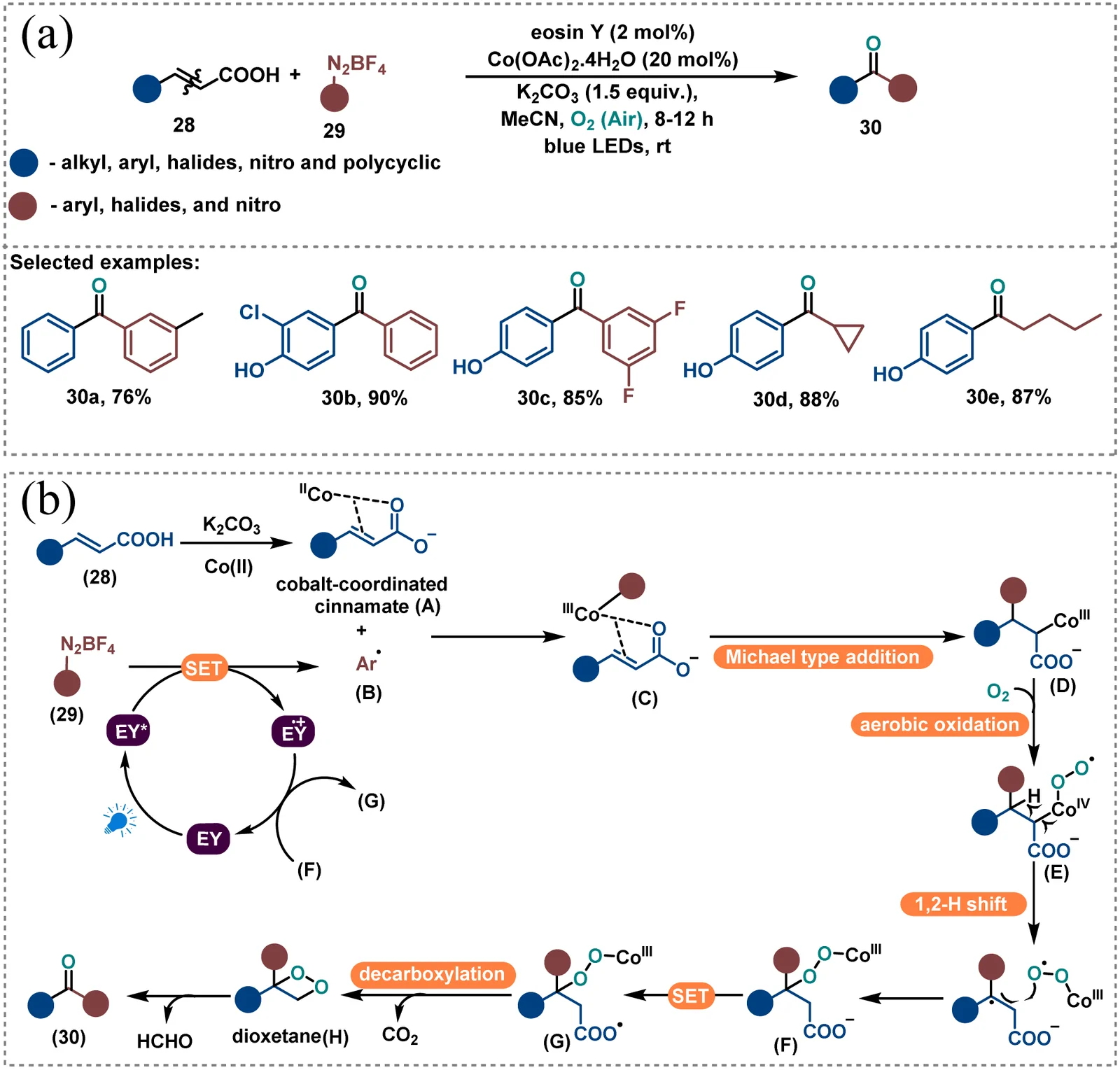
(a) Decarboxylative cross-coupling of α,β-unsaturated carboxylic acids with aryldiazonium salts. (b) Proposed mechanism for decarboxylative cross-coupling of α,β-unsaturated carboxylic acids with aryldiazonium salts.

Mechanistic studies suggest a photoredox radical pathway. Blue light irradiation activates eosin Y to its excited state, facilitating single-electron transfer (SET) to generate aryl radicals (B) from aryldiazonium salts (Scheme 10b). Concurrently, the α,β-unsaturated acid coordinates with cobalt(ii) to form a cinnamate–Co complex (A). The aryl radical adds to this intermediate *via* Michael-type addition, followed by aerobic oxidation and 1,2-hydride shift to generate an intermediate that undergoes SET oxidation and decarboxylation to form a dioxetane intermediate (H). This dioxetane decomposes with the loss of formaldehyde to furnish the final ketone product. The necessity of oxygen, the suppression of product formation by TEMPO, and the detection of oxetane and formic acid by HRMS all support this radical mechanism. This work presents an efficient and green approach to construct unsymmetrical ketones *via* cooperative visible-light and cobalt catalysis and extends the utility of diazonium salts in sustainable decarboxylative functionalization protocols.

Kushwaha and co-workers in 2024 developed a green and efficient visible-light-mediated, metal-free method for the synthesis of α,β-unsaturated ketones (chalcones) *via* direct hydroacylation of aromatic alkynes using C(sp^3^)–H functionalized methyl arenes (Scheme 11a).^85^ The reaction proceeds under mild conditions using eosin Y as an organic photocatalyst, air as the oxidant, and a MeCN : H_2_O (2 : 1) solvent system. This operationally simple and sustainable approach demonstrates high atom economy and excellent functional group tolerance, offering a valuable alternative to traditional metal-catalyzed hydroacylation strategies. The methodology shows broad compatibility with both aromatic alkynes and methyl arenes. Aromatic alkynes bearing electron-donating (*e.g.*, –Me, –OMe, –OH) and electron-withdrawing groups (*e.g.*, –NO_2_, –F, –Cl) furnished the desired chalcones in good to excellent yields (up to 89%). Substituted naphthyl, thiophenyl, and even cyclohexylacetylene were also tolerated, although internal or disubstituted alkynes were unreactive. On the methyl arene side, substrates with both electron-rich (*e.g.*, –Me, –OMe, –NH_2_) and electron-poor (*e.g.*, –F, –Cl, –CN, –NO_2_) substituents at *ortho*-, *meta*-, or *para*-positions delivered chalcones in high yields. The protocol was scalable and applicable to the late-stage functionalization of bioactive molecules.

**Scheme 11:**
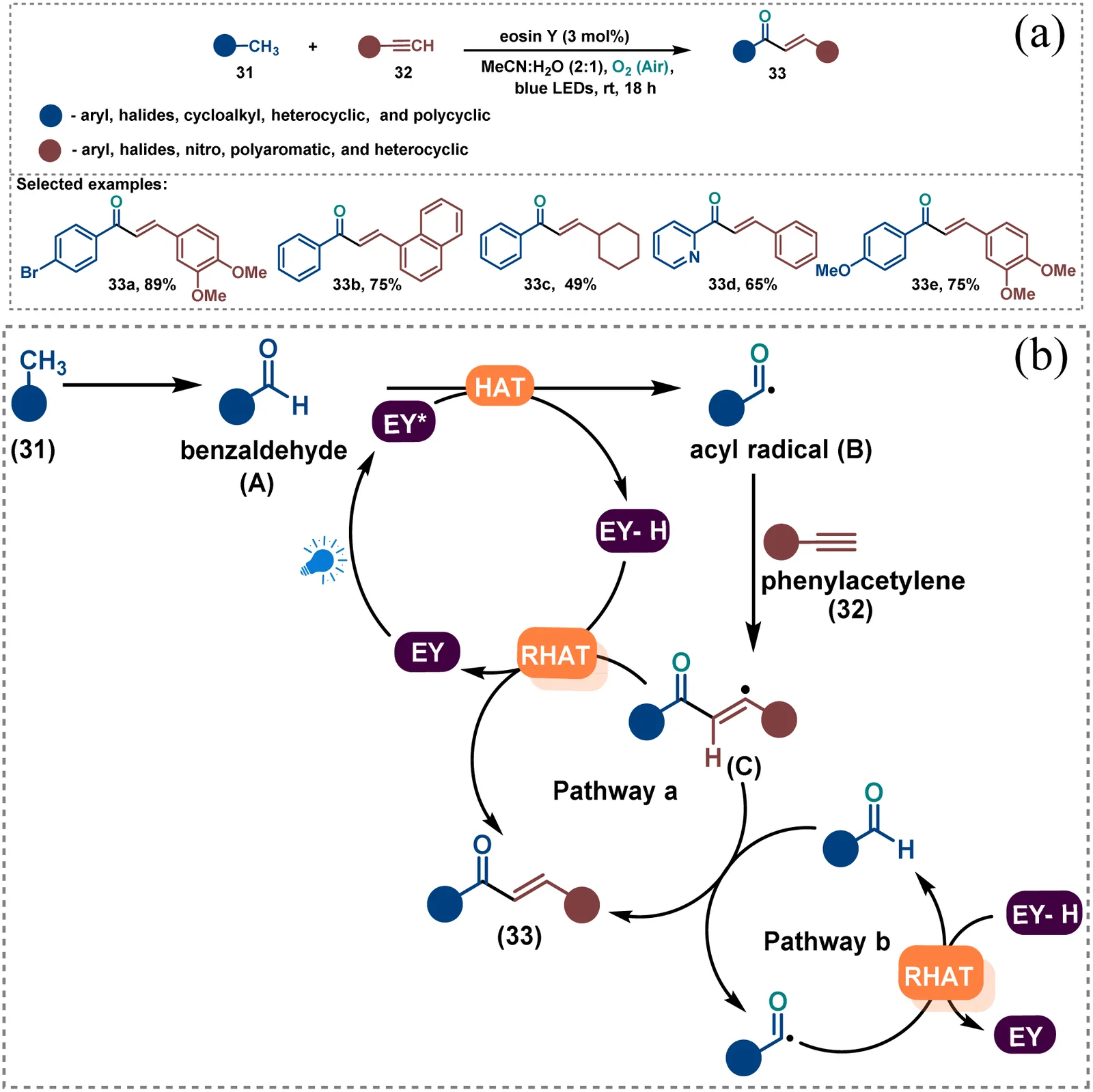
(a) Hydroacylation of aromatic alkynes *via* C(sp^3^)–H functionalization. (b) Proposed mechanism for hydroacylation of aromatic alkynes *via* C(sp^3^)–H functionalization.

Mechanistic studies, including radical trapping experiments (using TEMPO), UV-vis and fluorescence quenching analyses, and control reactions, support a photoinduced radical mechanism (Scheme 11b). Upon blue light irradiation, eosin Y is excited and facilitates hydrogen atom transfer (HAT) from methyl arenes to generate benzyl radicals, which are subsequently oxidized to acyl radicals (B). These radicals add to the alkyne, forming a new radical intermediate (C) that undergoes hydrogen atom transfer or recombination to deliver the chalcone product. The necessity of light, photocatalyst, and oxygen was confirmed through on/off light experiments and reactions under inert atmosphere. The formation of radical adducts and isolation of key intermediates supports the C–H activation-based radical pathway. This work offers the first demonstration of hydroacylation of alkynes *via* direct C(sp^3^)–H activation of methyl arenes in a metal-free photoredox setting and establishes a sustainable protocol for constructing synthetically and biologically important chalcones.

### 2.2. Photoredox catalyzed C–N oxygenative coupling reactions

In synthetic chemistry, there is a continued effort to develop effective and selective methods for easily forming carbon-nitrogen bonds due to their prevalence in natural products, functional materials, and pharmaceutical agents. A promising approach in achieving direct C–H amination involves employing photoredox catalysis. When subjected to photoirradiation, the photocatalyst aids in generating organoradical species through a single electron transfer (SET) pathway. These radicals can subsequently interact with various coupling partners to produce aminated products. Photocatalytic C–N bond formation includes oxidant-free amination and oxidative amination, typically initiated by the formation of a nitrogen radical cation, an aryl radical cation, or a nitrogen radical. Considering the importance of C–N bonds, herein we have provided some recent advances in oxidative C–N coupling reactions mediated by photocatalysis.^86–91^

Chen and co-workers in 2016 reported a visible-light-mediated three-component cyclization of 2*H*-azirines, alkynyl bromides, and molecular oxygen for the synthesis of functionalized oxazoles (Scheme 12a).^92^ The reaction utilizes an acridinium-based organic photocatalyst under blue LED irradiation at room temperature, proceeding under mild conditions with ambient air as a sustainable oxidant. This strategy provides a straightforward and metal-free route to construct oxazole frameworks, which are prominent in pharmaceuticals and natural products. The methodology displays broad substrate compatibility. Various 1-aryl-2-bromoacetylenes bearing electron-donating groups (*e.g.*, –Me, –Et, –OMe, –^*t*^Bu) and electron-withdrawing substituents (*e.g.*, –F, –Cl, –Br, –CF_3_) at *para*- and *meta*-positions reacted efficiently with 3-phenyl-2*H*-azirine to afford oxazoles in good yields (57–80%). Thiophenyl- and naphthyl-substituted alkynyl bromides were also compatible. The reaction was extended to substituted 2*H*-azirines bearing *para*-alkyl, halo, or trifluoromethyl groups, which cyclized with alkynyl bromides to furnish oxazoles in moderate to good yields (59–75%). *ortho*-Substituted substrates showed reduced reactivity due to steric hindrance.

**Scheme 12:**
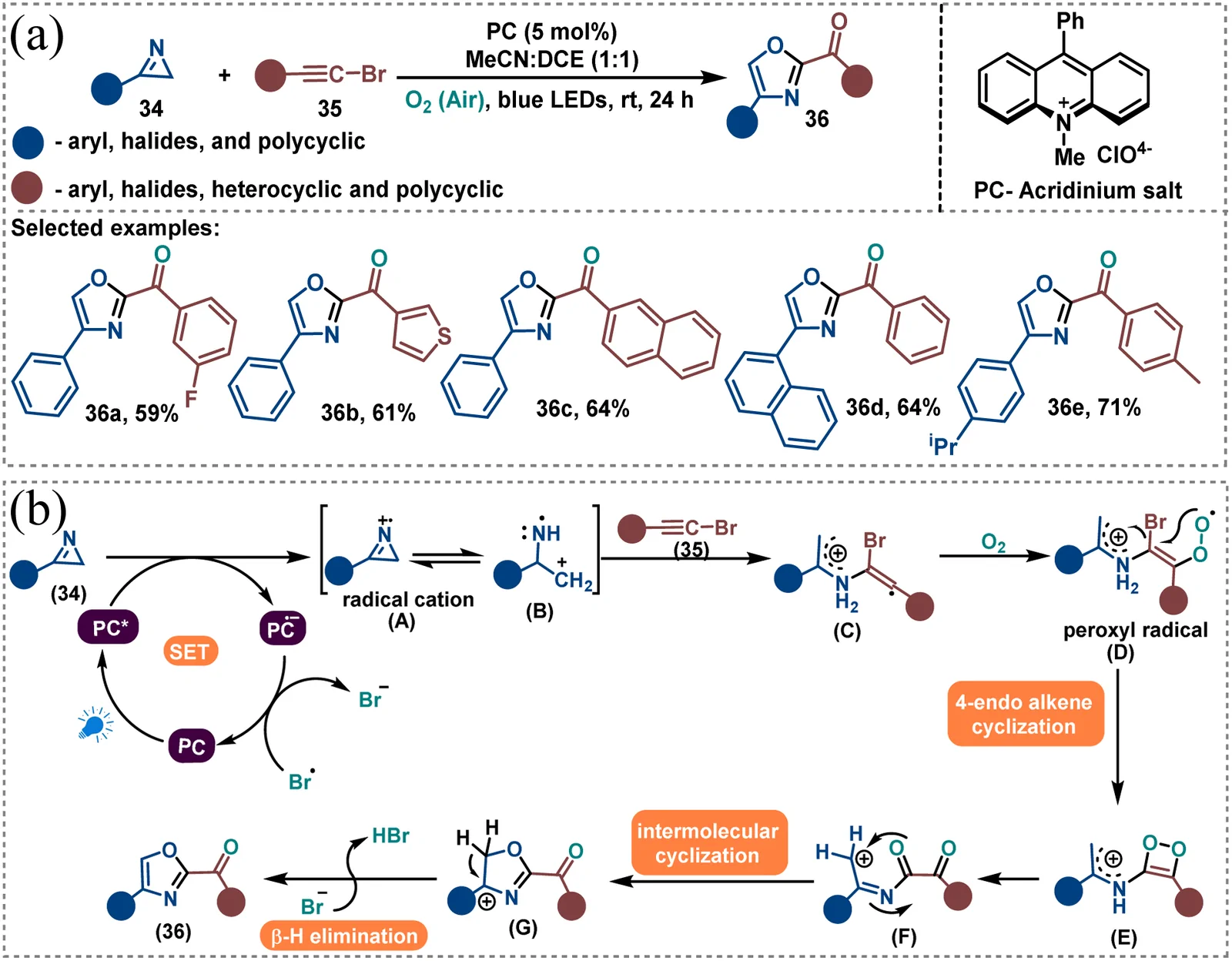
(a) Photoredox cyclization for the synthesis of substituted oxazoles. (b) Proposed mechanism for photoredox cyclization for the synthesis of substituted oxazoles.

Mechanistic studies suggest a radical-based pathway initiated by photoexcitation of the acridinium photocatalyst (PC*) under visible light (Scheme 12b). Single-electron transfer (SET) from 2*H*-azirine generates a radical cation (A), which adds to the alkynyl bromide to form an intermediate radical species (C). This radical couples with molecular oxygen to generate a peroxyl radical (D) that undergoes intramolecular cyclization to produce a vinyl peroxyl intermediate (E). Subsequent fragmentation and β-H elimination steps afford the oxazole product. The involvement of radical intermediates was confirmed by inhibition of the reaction in the presence of TEMPO and ^18^O_2_-labeling experiments demonstrated that the oxygen atom in the oxazole ring originates from molecular oxygen. This work introduces an efficient and environmentally benign protocol for constructing highly functionalized oxazoles under visible light, highlighting the utility of photoredox catalysis in multicomponent aerobic transformations.

Shukla and co-workers in 2019 developed a visible-light-mediated Ir(iii)-catalyzed protocol for the concomitant C3 oxidation and C2 amination of indoles using benzohydrazides under aerobic conditions (Scheme 13a).^93^ The reaction proceeds under mild conditions, utilizing [Ir(dtbbpy)(ppy)_2_]PF_6_ as the photocatalyst, tetrabutylammonium iodide (TBAI) as an additive, and white LED irradiation at room temperature. This method enables direct difunctionalization of indoles to generate oxindole derivatives with excellent regioselectivity and functional group tolerance. The method accommodates a broad range of *N*-substituted indoles and benzohydrazides. Both electron-donating and electron-withdrawing substituents on the indole nitrogen (*e.g.*, –Me, –Et, –^*n*^Pr, –^*n*^Bu, –^i^Pr, benzyl) and aromatic ring (*e.g.*, 5-Me, 5-Br, 7-F) were well tolerated, delivering the bifunctionalized oxindoles in moderate to good yields (65–87%). Benzohydrazides with various substituents (*e.g.*, *p*-Me, *p*-OMe, *p*-Br, *p*-CF_3_) reacted efficiently. The reaction also tolerated oxidation-sensitive functional groups such as allyl and benzylic moieties without degradation. However, alkyl hydrazides and sulfonyl hydrazides failed to yield the desired products.

**Scheme 13:**
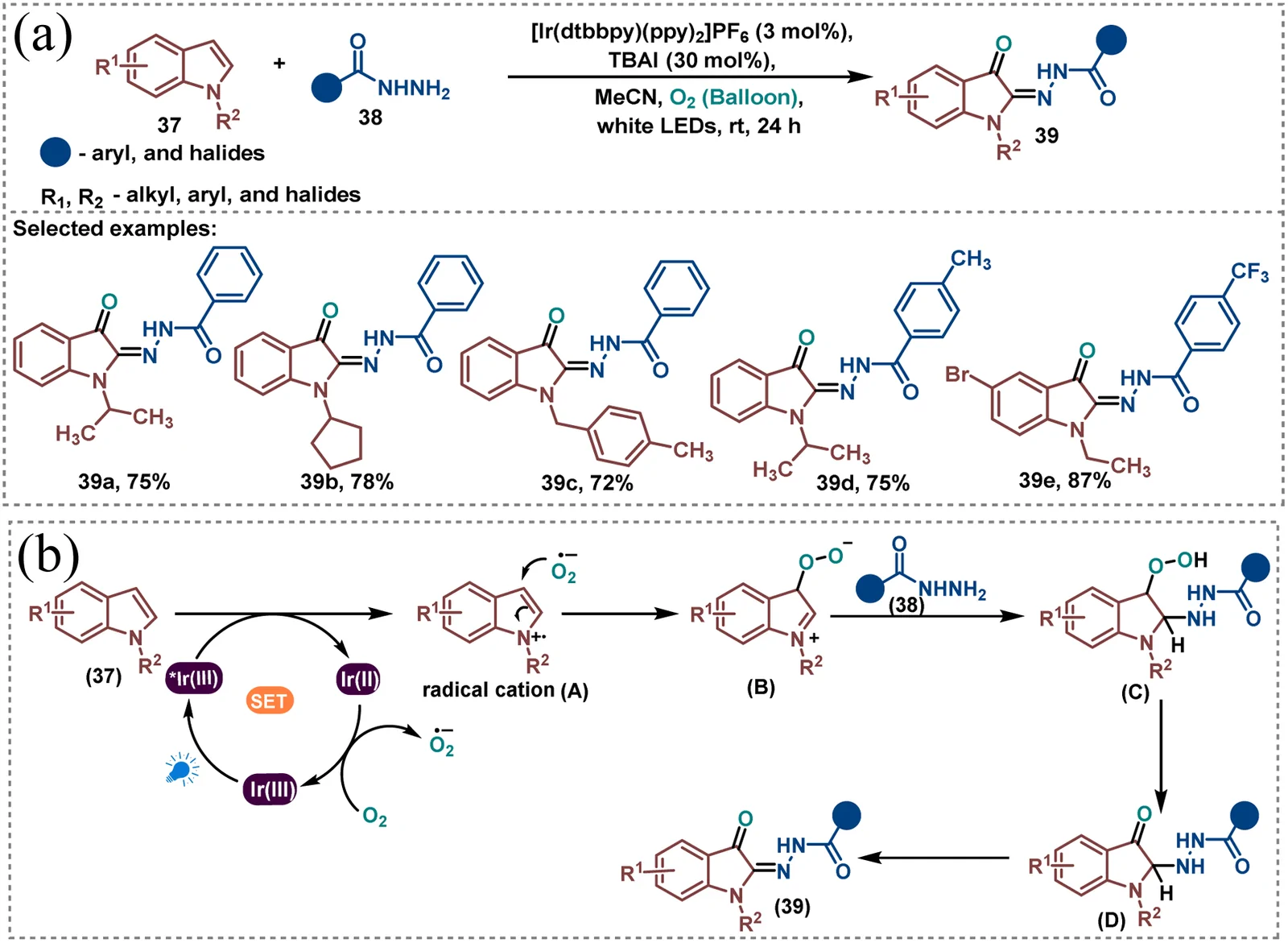
(a) Visible-light-induced Ir(iii)-catalyzed C3 oxidation and C2 amination of indoles. (b) Proposed mechanism for visible-light-induced Ir(iii)-catalyzed C3 oxidation and C2 amination of indoles.

Mechanistic investigations and DFT studies suggest a photoinduced radical pathway (Scheme 13b). Excitation of the Ir(iii) photocatalyst under visible light enables single-electron oxidation of the indole to generate a radical cation (A). The resulting intermediate undergoes regioselective attack by superoxide (O_2_˙^−^) at C3 to form a peroxy intermediate (B). Subsequent nucleophilic attack by benzohydrazide at C2 leads to a bifunctionalized intermediate (C), which eliminates water and undergoes further oxidation to furnish the final oxindole product. Control experiments with TEMPO inhibited the reaction, confirming the involvement of radical intermediates, and ^18^O_2_-labeling studies verified that molecular oxygen is the source of the incorporated oxygen. This strategy provides a mild, operationally simple, and regioselective method for constructing highly functionalized oxindoles, demonstrating the utility of visible-light photoredox catalysis in heterocycle functionalization.

Hwang and co-workers in 2020 developed a visible-light-driven, copper-catalyzed protocol for the regioselective acetamidation of terminal alkynes with arylamines under mild conditions (Scheme 14a).^94^ The transformation proceeds with inexpensive CuCl as the catalyst, K_2_CO_3_ as a base, and ambient air as the terminal oxidant, in a MeCN : MeOH (1 : 1) solvent system under blue LED irradiation at room temperature. The process avoids the need for external ligands or protecting groups and demonstrates excellent scalability and green chemistry metrics. The method tolerates a wide variety of arylamines and terminal alkynes. Electron-rich anilines efficiently afforded the desired acetamides in high yields (up to 87%). Notably, unprotected 2-aminophenol and anthranilic acids participated without pre-functionalization, delivering acetamides and, in the case of anthranilic acids, simultaneous amide and ester functionalities. Diverse terminal alkynes, including aryl-, heteroaryl-, and aliphatic alkynes, were compatible, affording products in moderate to excellent yields. Heteroaryl alkynes, such as ethynylthiophenes and carbazole alkynes, also reacted smoothly under the oxidative conditions.

**Scheme 14:**
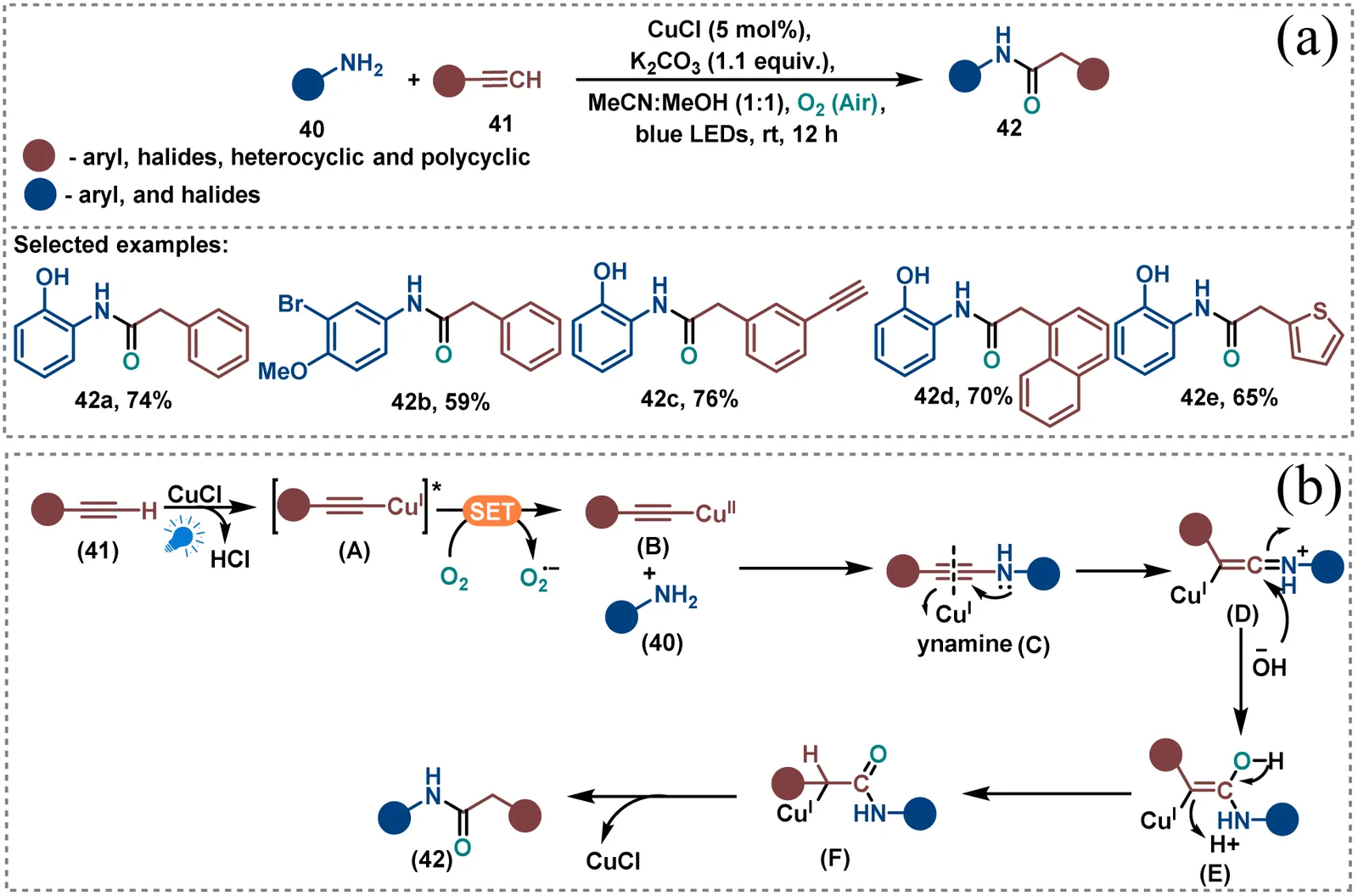
(a) Copper-catalyzed regioselective acetamidation of terminal alkynes by arylamines. (b) Proposed mechanism for copper-catalyzed regioselective acetamidation of terminal alkynes by arylamines.

Mechanistic investigations indicate a photoredox radical pathway (Scheme 14b). *In situ* generated Cu(i)-phenylacetylide absorbs visible light and reaches a long-lived triplet excited state (A). This species undergoes single-electron transfer (SET) to molecular oxygen, generating a Cu(ii)-phenylacetylide (B) and a superoxide radical anion. The Cu(ii) complex reacts with arylamines *via* nucleophilic addition and subsequent oxidative processes to afford highly reactive Cu(iii)-intermediates. Reductive elimination yields the ynamine intermediates (C), which cyclize to produce the final acetamides. Control experiments with TEMPO confirmed the involvement of radical intermediates, and ^18^O_2_-labeling studies revealed distinct oxygen incorporation pathways depending on the substrate. This sustainable method enables efficient and regioselective formation of amides and esters under photoredox conditions, with applications demonstrated in the synthesis of bioactive inhibitors such as BACE-1 and PDE4.

Xiao and co-workers in 2021 reported a visible-light-promoted, photocatalytic strategy for synthesizing perfluoroalkyl amides and esters from amines and alcohols using perfluoroalkyl iodides (Scheme 15a).^95^ The method employs *fac*-[Ir(ppy)_3_] as the photocatalyst and operates under mild, metal-free conditions using ambient air as the oxidant. This work highlights the significance of excited-state redox potentials in photocatalyst design for efficient activation of perfluoroalkyl halides. The methodology demonstrates wide applicability for both amidation and esterification. A variety of aromatic and aliphatic amines reacted smoothly with perfluoroalkyl iodides to afford perfluoroalkyl amides in moderate to excellent yields. Aliphatic amines generally gave higher yields than aromatic amines due to reduced competitive quenching of the photocatalyst. For esterification, primary and secondary alcohols delivered moderate yields of perfluoroalkyl esters, while tertiary alcohols gave only trace amounts of products. Notably, phenol and benzyl alcohol failed to produce esters, likely due to their radical-scavenging properties. The amidation reactions were more efficient than esterifications, with fewer by-products such as perfluoroalkanoic acids observed.

**Scheme 15:**
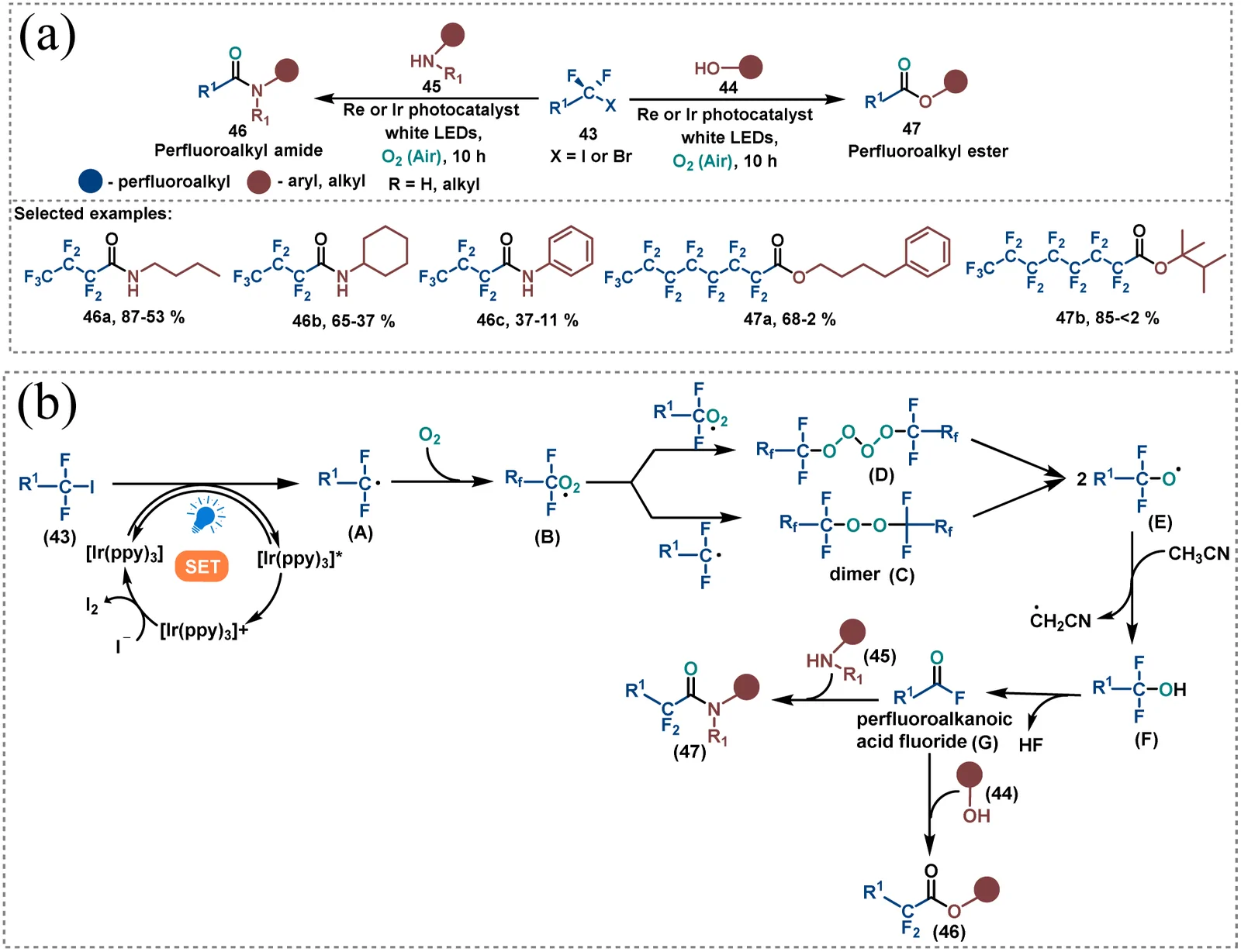
(a) Amidation and esterification with perfluoroalkyl iodide. (b) Proposed mechanism for amidation and esterification with perfluoroalkyl iodide.

Mechanistic investigations and DFT calculations support a radical pathway initiated by oxidative quenching of photoexcited *fac*-[Ir(ppy)_3_] by perfluoroalkyl iodides, generating perfluoroalkyl radicals (A) (Rf˙) (Scheme 15b). These radicals react with oxygen to form perfluoroalkyl peroxyl intermediates (B). For amidation, the peroxyl radicals convert into perfluoroacyl fluorides, which are subsequently attacked by amines to produce amides. In esterification, analogous perfluoroacyl intermediates react with alcohols to form esters, though with lower efficiency due to competing formation of perfluoroalkanoic acids. Emission quenching experiments and radical trapping studies confirmed the involvement of Rf˙ radicals and supported the proposed pathways. This work provides a green and efficient approach for incorporating perfluoroalkyl groups into amides and esters under visible light and highlights the importance of photocatalyst redox properties in tuning reactivity for perfluoroalkyl halides.

Manhas and co-workers in 2021 reported a sustainable, metal-free visible-light-driven protocol for synthesizing α-ketoamides through sequential oxidative amidation–diketonization of terminal alkynes (Scheme 16a).^96^ This transformation uses rose bengal as an organic photocatalyst, thiophenol as a hydrogen atom donor, and atmospheric oxygen as the oxidant under compact fluorescent lamp (CFL) irradiation at room temperature. The method provides access to α-ketoamides, key intermediates in bioactive molecules and heterocycle synthesis, under mild and environmentally friendly conditions. The methodology demonstrated broad substrate compatibility with various terminal alkynes and secondary amines. Phenylacetylenes bearing electron-donating groups (*e.g.*, –Me, –Et, –^*t*^Bu, –OMe) afforded α-ketoamides in good yields (65–78%), while those with electron-withdrawing substituents (*e.g.*, –F, –Cl, –Br, –CF_3_) delivered slightly lower yields (61–70%). Halogenated and disubstituted phenylacetylenes were also effective coupling partners. Secondary cyclic amines such as pyrrolidine, piperidine, and morpholine, as well as acyclic secondary amines like diethylamine, furnished the products in moderate to good yields (62–78%). In contrast, primary amines and aliphatic alkynes were unreactive under the optimized conditions, possibly due to instability of aliphatic vinylic radical intermediates.

**Scheme 16:**
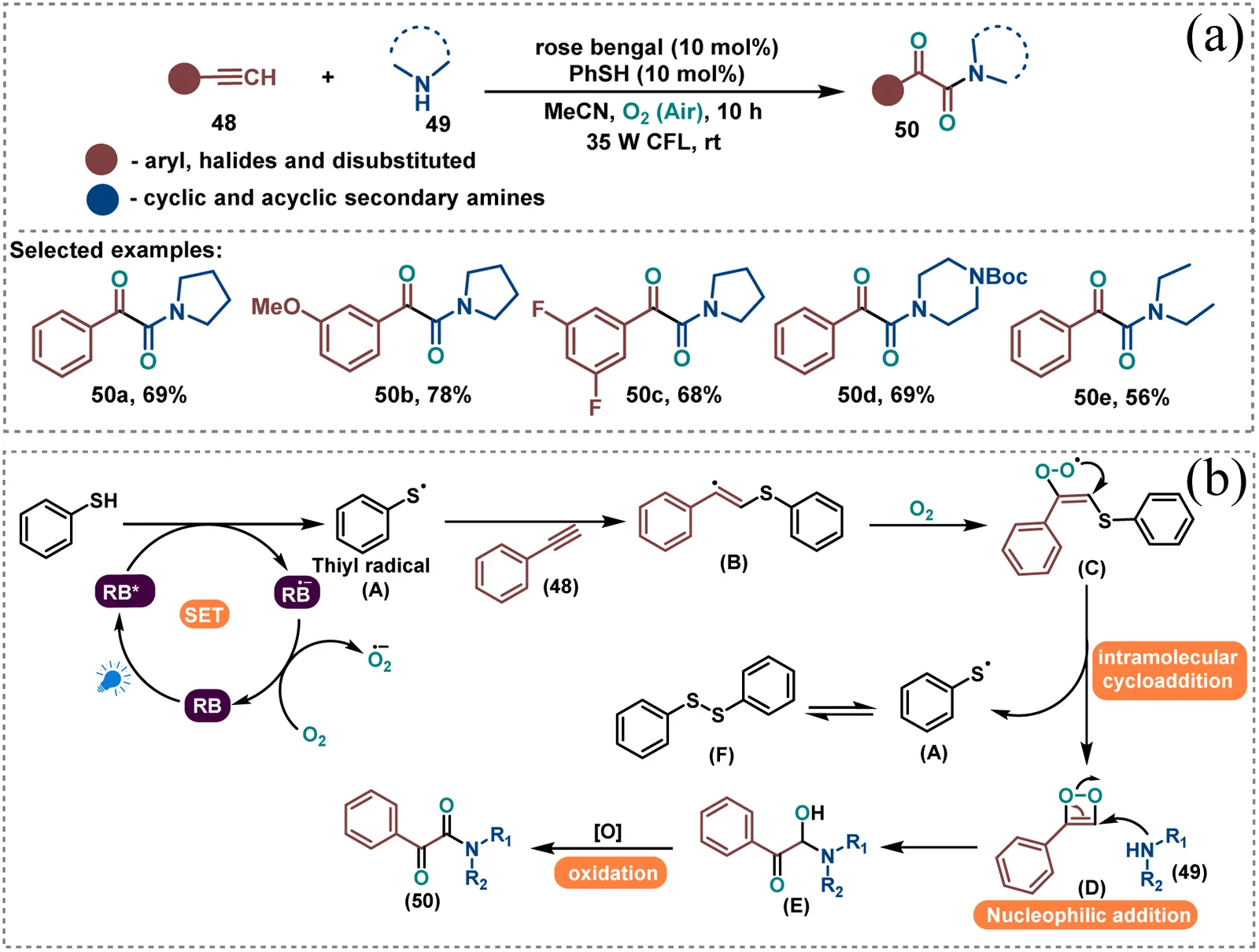
(a) Oxidative amidation–diketonization of terminal alkynes. (b) Proposed mechanism for oxidative amidation–diketonization of terminal alkynes.

Control experiments and photophysical studies support a radical-based mechanism initiated by visible light excitation of rose bengal (RB) (Scheme 16b). Upon irradiation, RB is photoexcited and undergoes reductive quenching with thiophenol to generate a thiyl radical (A). The thiyl radical adds to the terminal alkyne, forming a vinylic radical (B) that is subsequently oxidized by O_2_ to generate a peroxy intermediate (C). This species undergoes intramolecular cyclization to form a dioxetane intermediate (D), which, upon nucleophilic attack by an amine and subsequent oxidation, yields the α-ketoamide. The reaction was suppressed in the presence of radical scavengers (*e.g.*, TEMPO), confirming the involvement of radical intermediates. Stern–Volmer quenching and UV-vis studies further substantiated that thiophenol plays a dominant role in quenching excited RB and driving the single-electron transfer (SET) process. This work provides a mild, scalable, and metal-free alternative for constructing α-ketoamide frameworks and highlights the potential of visible-light photoredox catalysis in developing green synthetic methodologies.

Kishor, Prabhakar, and Singh in 2023 reported an efficient visible-light-induced, eosin Y catalyzed oxidative amination of 2-bromoacetophenones with amines to synthesize α-ketoamides under mild and metal-free conditions (Scheme 17a).^97^ The protocol utilizes atmospheric oxygen as the oxidant and blue LED irradiation to drive the photoredox process, affording a wide variety of α-ketoamides with high functional group tolerance and scalability. The method shows broad substrate compatibility. Various 2-bromoacetophenones with electron-donating (*e.g.*, –Me, –OMe) and electron-withdrawing (*e.g.*, –F, –Cl, –Br, –NO_2_, –CN, –SO_2_Me) groups on the aromatic ring reacted efficiently. Cyclic secondary amines such as pyrrolidine, morpholine, and piperidine afforded the corresponding α-ketoamides in high yields (73–86%). Acyclic secondary amines (*e.g.*, dimethylamine) and primary amines (*e.g.*, *n*-butylamine) also participated successfully, yielding the desired products in 72–82%. The method tolerated different leaving groups (*e.g.*, –I, –OTs) on phenacyl substrates and was extended to methyl 2-bromoacetate, which gave the corresponding ketoamide in 81% yield. The scalability of the reaction was demonstrated by a gram-scale synthesis, delivering the product with consistent efficiency.

**Scheme 17:**
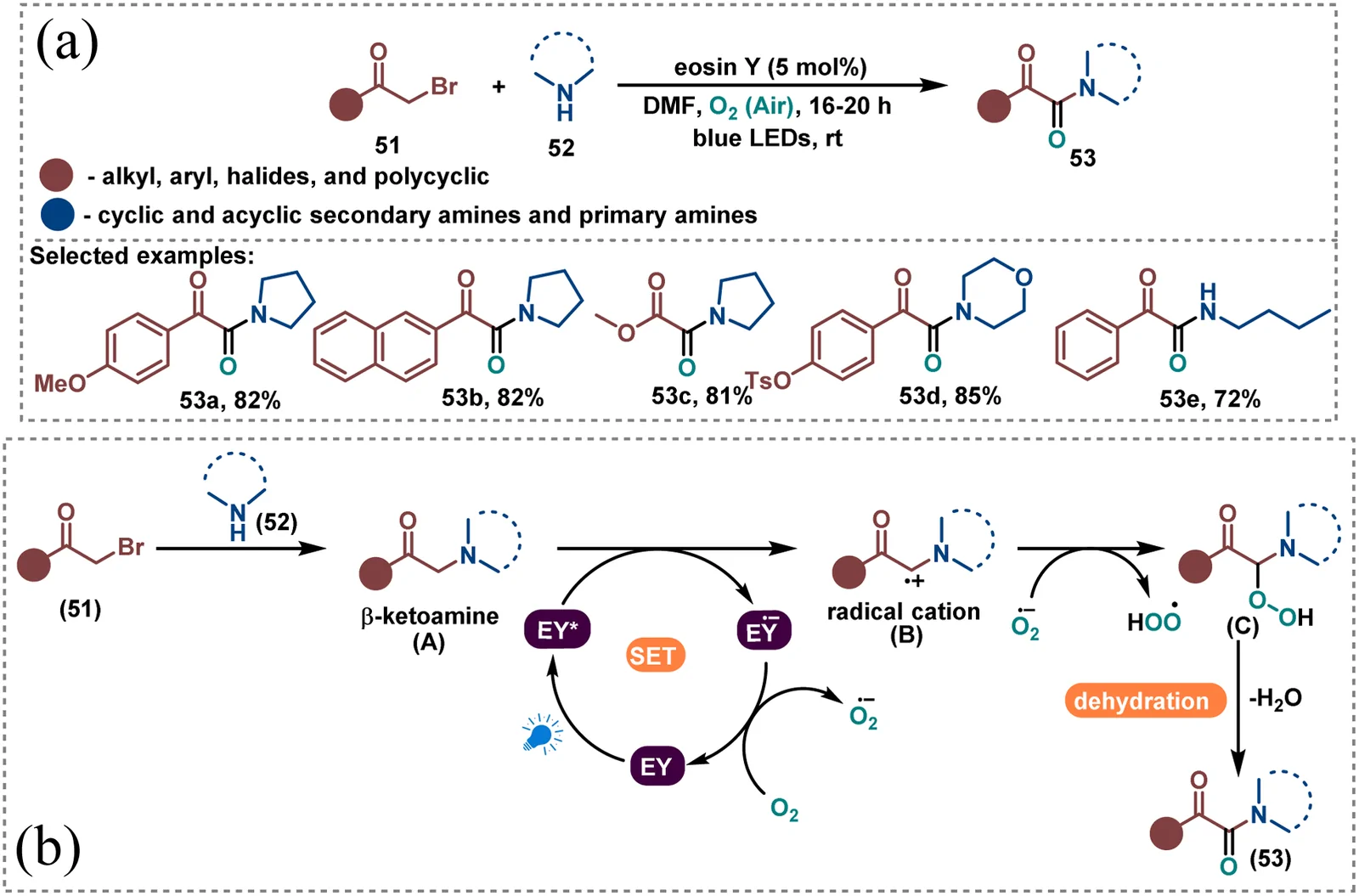
(a) Oxidative amination of 2-bromoacetophenones. (b) Proposed mechanism for oxidative amination of 2-bromoacetophenones.

Mechanistic studies indicate a radical-mediated oxidative pathway (Scheme 17b). Initially, the nucleophilic substitution of bromide by the amine generates a β-ketoamine intermediate (A). Upon visible light irradiation, eosin Y is photoexcited (EY*), which undergoes single-electron transfer (SET) with the β-ketoamine, generating a radical cation (B). This species is trapped by superoxide radical anion (O_2_˙^−^), produced *in situ via* eosin Y photoredox cycling, forming a radical intermediate. Subsequent dehydration delivers the α-ketoamide product. Control experiments confirmed the essential roles of visible light, photocatalyst, and molecular oxygen. The reaction was suppressed in the presence of radical scavengers (TEMPO, BHT), supporting the radical mechanism. This operationally simple and environmentally friendly protocol provides a sustainable route to synthetically valuable α-ketoamides, highlighting the utility of photoredox catalysis in green C–N bond-forming transformations.

Das and co-workers in 2023 developed a sustainable and operationally simple method for synthesizing α-ketoamides through a visible-light-driven, metal- and photocatalyst-free tandem amidation process (Scheme 18a).^98^ The reaction uses molecular oxygen both as an oxidant and as a source of oxygen in the amide functionality. Under mild conditions (9 W CFL, room temperature), phenacyl bromides react with primary amines in the presence of K_2_CO_3_ to afford α-ketoamides with high regioselectivity and functional group tolerance. The methodology displays broad compatibility with various phenacyl bromides and amines. Electron-rich aromatic amines (*e.g.*, –Me, –OMe, –^*t*^Bu) gave excellent yields (up to 94%), while electron-withdrawing groups (*e.g.*, –CF_3_, –NO_2_, –CN) on the aromatic ring delivered slightly reduced yields (69–75%). *ortho*-, *meta*-, and *para*-Substituted anilines, disubstituted anilines, and heteroaromatic amines were all effective, although pyridyl and pyrimidyl amines exhibited poor reactivity. The method also tolerated aliphatic amines (*e.g.*, butyl, cyclohexyl, adamantyl), which provided the desired α-ketoamides in good yields. The reaction could be scaled up to gram quantities without loss of efficiency.

**Scheme 18:**
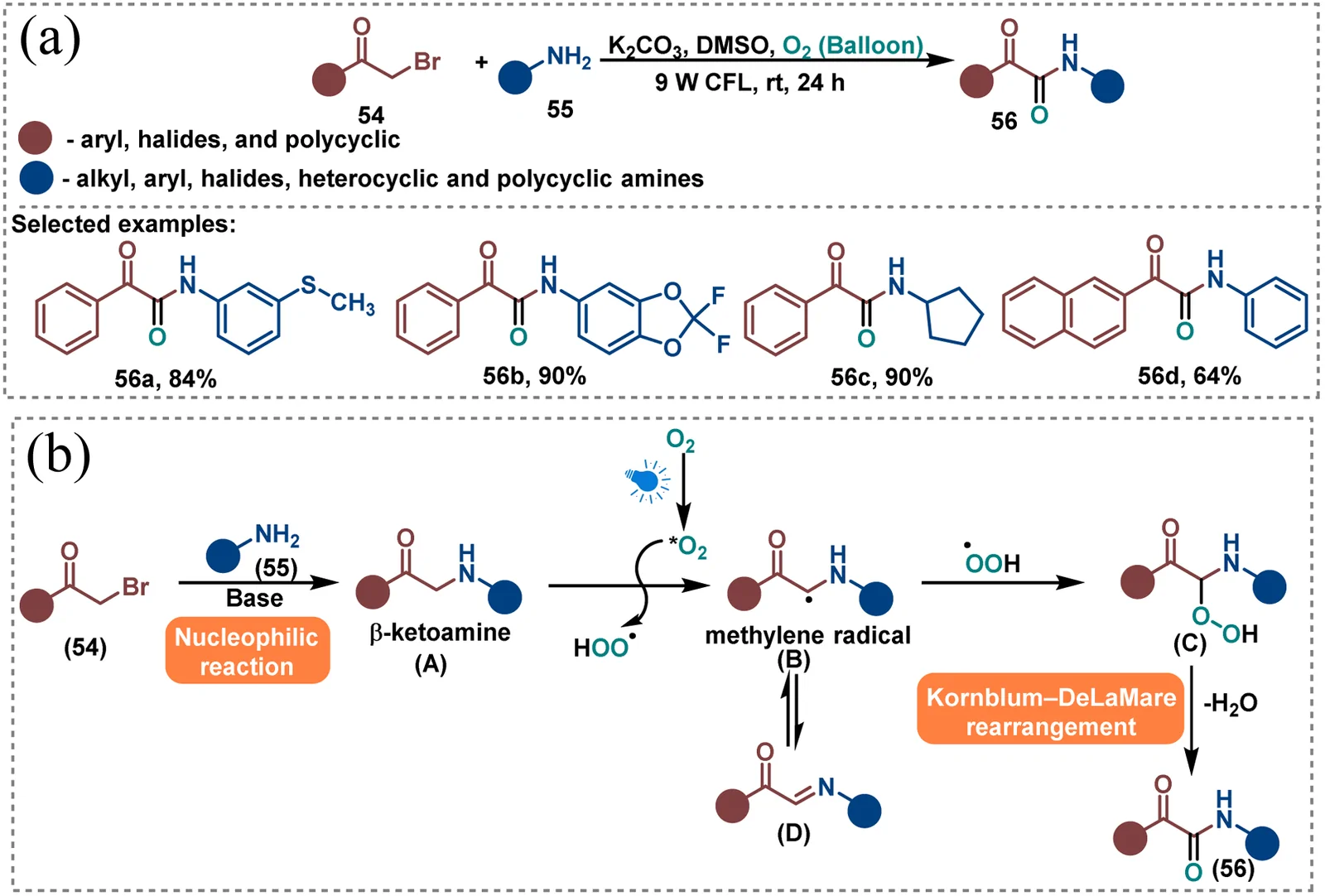
(a) Metal- and photocatalyst-free synthesis of α-ketoamides *via* base-promoted tandem amidation. (b) Proposed mechanism for metal- and photocatalyst-free synthesis of α-ketoamides *via* base-promoted tandem amidation.

Mechanistic investigations suggest a tandem radical pathway (Scheme 18b). Initially, the amine nucleophilically displaces bromide from phenacyl bromide to form a β-ketoamine intermediate (A). Subsequent oxidative dehydrogenation under visible light and molecular oxygen generates a methylene radical species (B). This radical couples with peroxide radicals derived from photoexcited singlet oxygen, leading to a radical–radical intermediate (C) that undergoes Kornblum–DeLaMare rearrangement to afford the α-ketoamide. Radical trapping experiments (using BHT) and ^18^O_2_-labeling studies confirmed the radical nature of the reaction and the incorporation of molecular oxygen into the carbonyl group. This green and efficient method provides a sustainable alternative for accessing α-ketoamides, avoiding the use of transition metals, stoichiometric oxidants, or photocatalysts, and producing only water as a byproduct.

Kushwaha *et al.* in 2023 developed an environmentally benign and operationally simple protocol for the synthesis of arylhydrazones through visible-light-induced C–N cross-coupling between indoles or methylarenes and arylhydrazines (Scheme 19a).^99^ The transformation proceeds under metal-free conditions using eosin Y as an organic photocatalyst, an EtOH : H_2_O (2 : 1) mixture as a green solvent, and atmospheric oxygen as the sole oxidant. This methodology delivers the desired products in good to excellent yields, exhibits broad functional group tolerance, and obviates the need for pre-functionalized substrates or harsh reaction conditions. The methodology accommodates electron-rich and electron-deficient indoles (*e.g.*, –Cl, –Br, –F, –NO_2_, –Me, –OMe), *N*-substituted indoles, and diverse arylhydrazines, including heteroaryl variants, giving 82–93% yields. Methylarenes bearing halogens, nitro, methoxy, naphthyl, and heteroaryl substituents also react efficiently, affording 85–93% yields across a wide range of arylhydrazines.

**Scheme 19:**
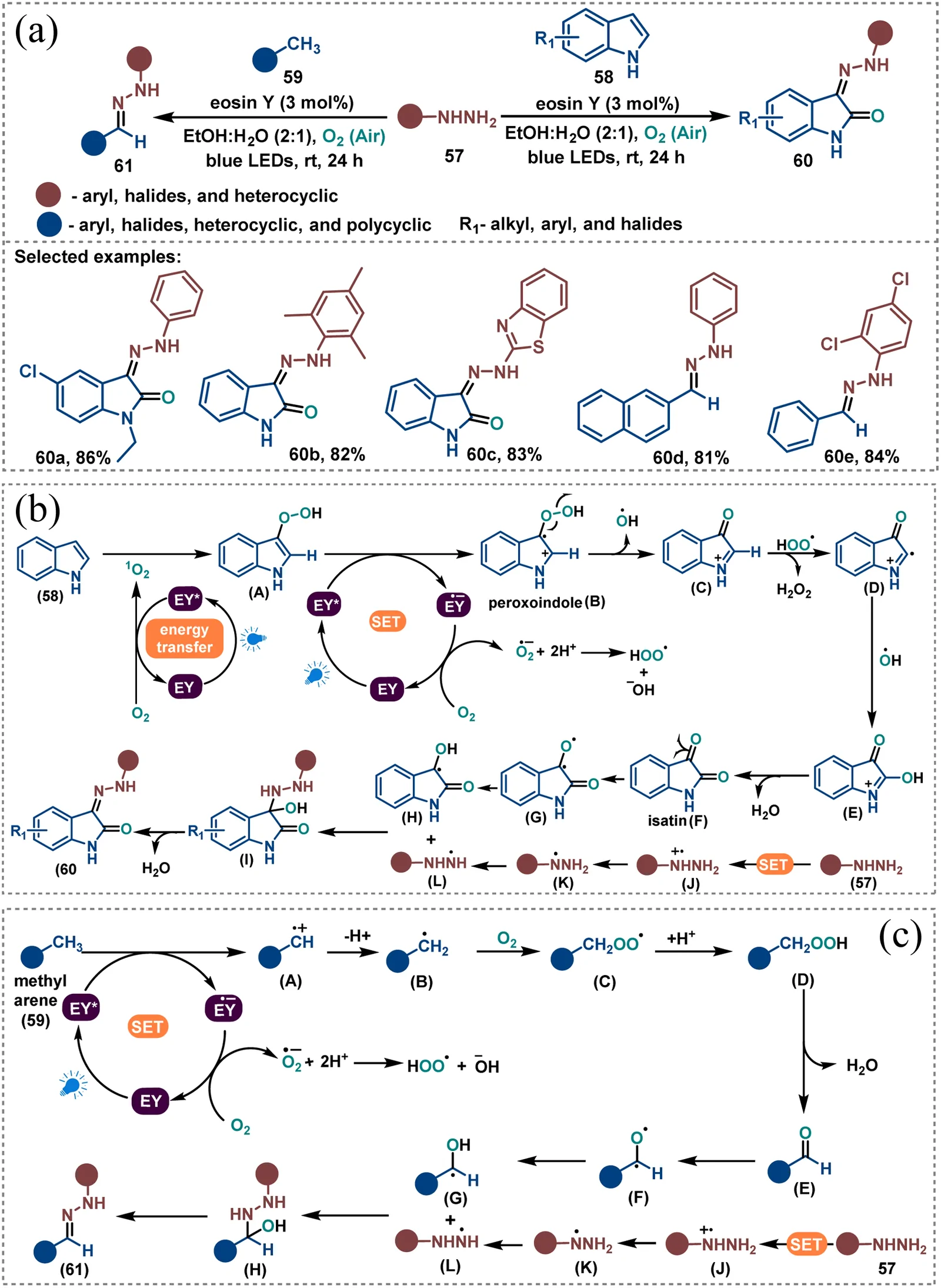
(a) C–N cross-coupling *via* C(sp^2^)–H and C(sp^3^)–H functionalization. (b) Proposed mechanism for C–N cross-coupling *via* C(sp^2^)–H functionalization. (c) Proposed mechanism for C–N cross-coupling *via* C(sp^3^)–H functionalization.

Mechanistic studies indicate a photoredox radical pathway (Scheme 19b and c). In the C(sp^2^)–H functionalization of indoles, photoexcited eosin Y generates singlet oxygen, which oxidizes the indole to peroxo intermediates that fragment into reactive electrophiles, subsequently coupling with phenylhydrazine-derived radicals to furnish hydrazones. In the C(sp^3^)–H functionalization of methylarenes, eosin Y promotes single-electron oxidation to a radical cation, which deprotonates to a benzyl radical; this reacts with oxygen to yield peroxo intermediates that dehydrate to aldehydes, which then condense with arylhydrazines. Radical inhibition experiments and ^18^O_2_-labeling studies confirm the oxygen- and radical-mediated nature of the process.

Sun and colleagues in 2026 used pyridine-based organic dye photocatalysts in mild circumstances to create an effective and durable photocatalytic process for the oxidative coupling of benzylamines (Scheme 20a).^100^ The approach makes use of donor–acceptor (D–A) chromophore complexes, namely dyes produced from triphenylamine (TPA-by) and tetraphenylethylene (TPE-by), which have tunable electronic structures and advantageous photophysical characteristics. With atmospheric oxygen serving as the only oxidant, the procedure produces imines at exceptional yields of up to 97% under UV (365 nm LED) irradiation in acetonitrile and ambient air. Interestingly, the reaction proceeds without the use of additives, demonstrating its ease of use and eco-friendliness.

**Scheme 20:**
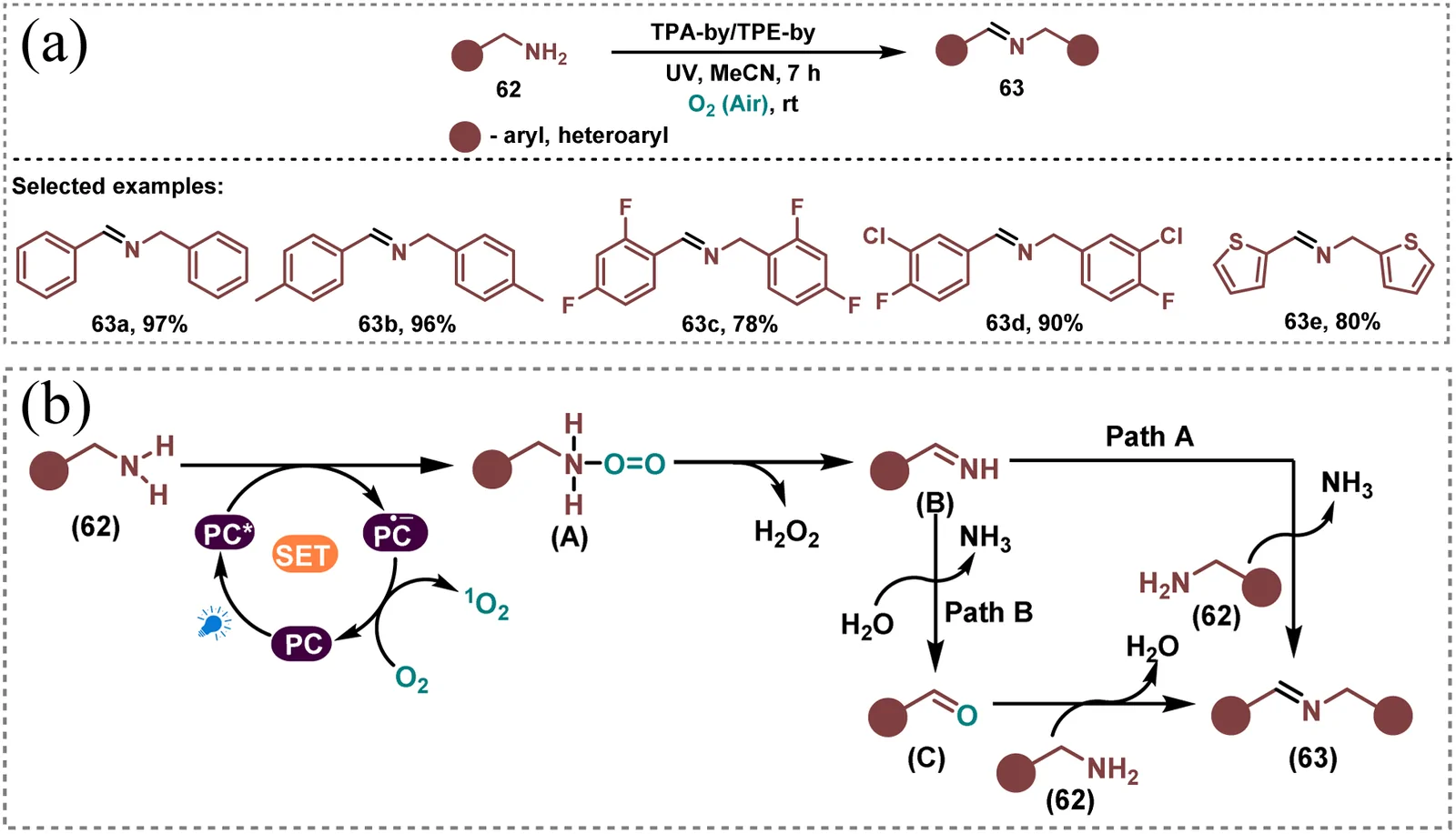
(a) Photocatalytic process for the oxidative coupling of benzylamines. (b) Proposed mechanism of photocatalytic process for the oxidative coupling of benzylamines.

With little electronic bias, a wide variety of benzylamine derivatives with both electron-donating and electron-withdrawing substituents were well tolerated and produced the intended products in good to excellent yields (72–97%). TPA-by outperformed the other catalysts because of its better charge separation efficiency, shorter band gap, and increased π-conjugation. Additionally, both photocatalysts showed outstanding recyclability, maintaining about 90% activity across several cycles.

An energy-transfer mechanism involving reactive oxygen species (Scheme 20b), specifically singlet oxygen (^1^O_2_), was discovered through mechanistic investigations such as quenching and EPR tests. Molecular oxygen is activated by the photoexcited catalyst to produce ^1^O_2_, which facilitates hydrogen abstraction and the subsequent synthesis of imines. Light, photocatalyst, and oxygen are necessary for the transformation, as demonstrated by control studies.

Overall, this method provides a mild, metal-free, and efficient approach for imine synthesis, although limitations such as UV-light dependence and restricted substrate scope remain.

By combining sulfoximines with α-bromomethyl aryl ketones, Nandi and colleagues in 2026 created a gentle and metal-free photocatalytic method for the synthesis of α-keto-*N*-acyl sulfoximines (Scheme 21a).^101^ The technique uses molecular oxygen as the terminal oxidant and eosin Y as a cheap organic photocatalyst under green LED irradiation (525 nm). Its sustainability and ease of use are highlighted by the transformation's notable ability to occur under ambient settings without the need of harsh reagents or external oxidants.

**Scheme 21:**
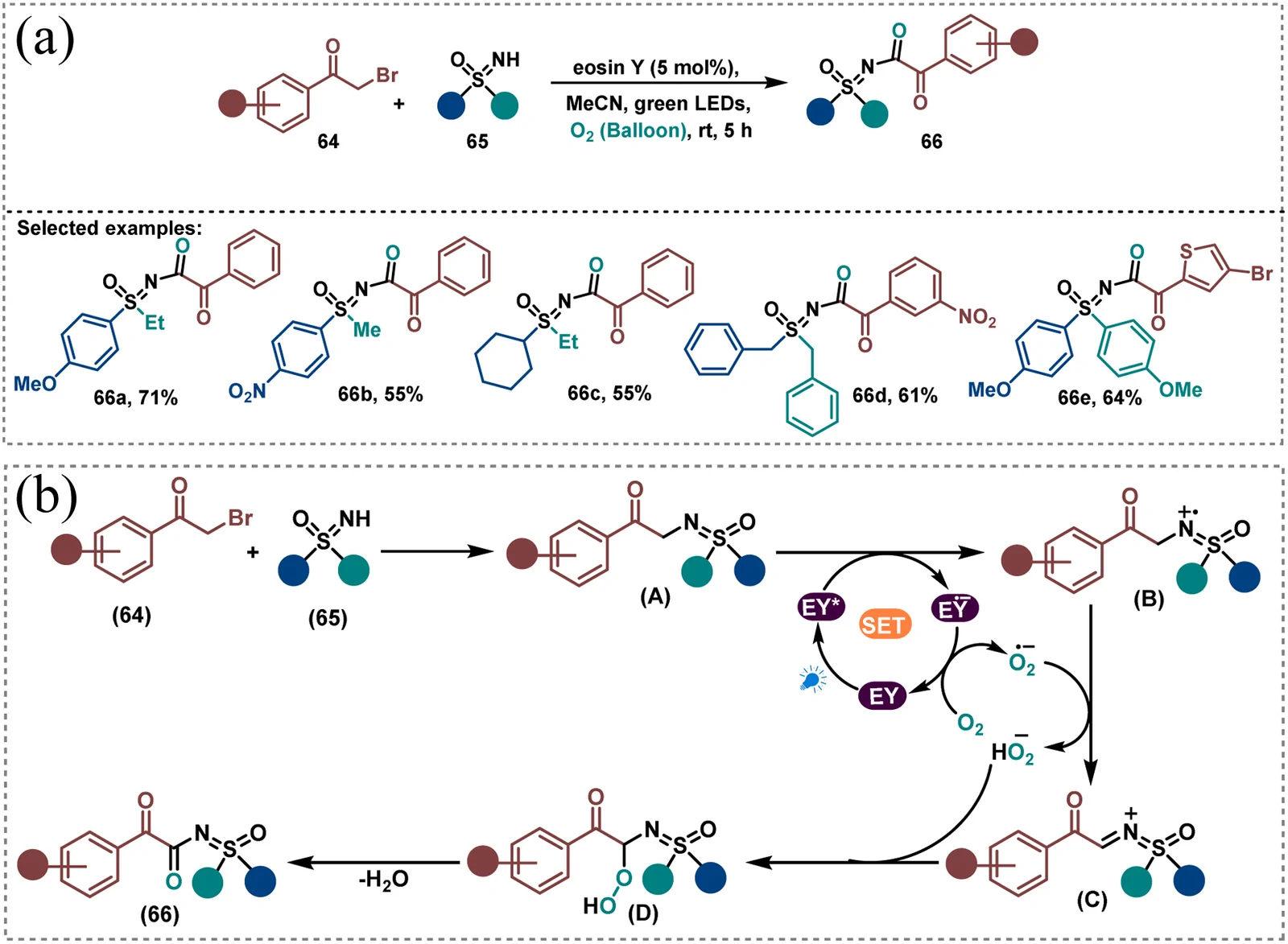
(a) Photocatalytic method for the synthesis of α-keto-*N*-acyl sulfoximines. (b) Proposed mechanism of photocatalytic method for the synthesis of α-keto-*N*-acyl sulfoximines.

*S*-Aryl, *S*-alkyl, and *S*,*S*-dialkyl derivatives were among the several sulfoximines that were well tolerated and produced the corresponding products in moderate to good yields. Similar to this, a variety of α-bromomethyl aryl ketones with heteroaryl groups and electron-donating and electron-withdrawing substituents successfully took part in the transition. However, it was discovered that aliphatic substrates, including α-bromomethyl alkyl ketones, were unreactive, suggesting certain substrate restrictions. Gram-scale synthesis and additional synthetic modifications of the products showed the protocol's practical usefulness.

The involvement of a radical pathway was validated by mechanistic investigations, such as radical trapping experiments using TEMPO and BHT (Scheme 21b). When eosin Y is excited by visible light, photoinduced electron transfer occurs, producing a reactive radical species that then combines with the intermediate generated from sulfoximine. Superoxide species produced *in situ* capture the resultant radical intermediate, which is then dehydrated to produce the required α-keto-*N*-acyl sulfoximine product. The critical functions of light, photocatalyst, and oxygen in the reaction were verified by control tests. Overall, this technique offers a metal-free, efficient, and environmentally friendly way to create useful sulfoximine frameworks through the creation of C–N and C–O bonds; nevertheless, substrate scope restrictions and occasionally low yields still exist.

### 2.3. Photoredox catalyzed C–S/C–O oxygenative coupling reactions

The synthesis of C–S bonds holds significant synthetic importance due to the abundance of sulfur-containing natural products, drugs, and agrochemicals, as well as the growing relevance of sulfur-containing compounds in material and polymer chemistry. Organic compounds containing sulfur are frequently utilized in many different areas of chemistry. Due to the growing number of authorized organosulfur-based medications, numerous investigations have focused on incorporating sulfur functional groups into organic compounds. Photoredox catalysis has garnered considerable interest in building structurally diverse and intricate organic frameworks due to its inherent attributes such as high efficiency, environmental friendliness, sustainability, renewability, cost-effectiveness, and ease of use. Considering these foundations, we have opted to outline the progress made in developing protocols for visible-light-induced oxidative coupling to synthesize C–S bonds.^88,102–106^

Liu *et al.* in 2019 reported a catalyst-free, visible-light-driven oxidative coupling between aryldiazo sulfones and thiols, enabling the efficient synthesis of unsymmetrical sulfoxides under ambient air (Scheme 22a).^107^ The reaction requires only blue LED irradiation at room temperature, without any photocatalyst, metal additives, or external oxidants, making it a highly sustainable protocol. A broad range of thiols-including electron-rich, electron-poor, sterically hindered aryl thiols as well as heteroaryl and aliphatic thiols-react smoothly with diverse aryldiazo sulfones to afford sulfoxides in moderate to good yields.

**Scheme 22:**
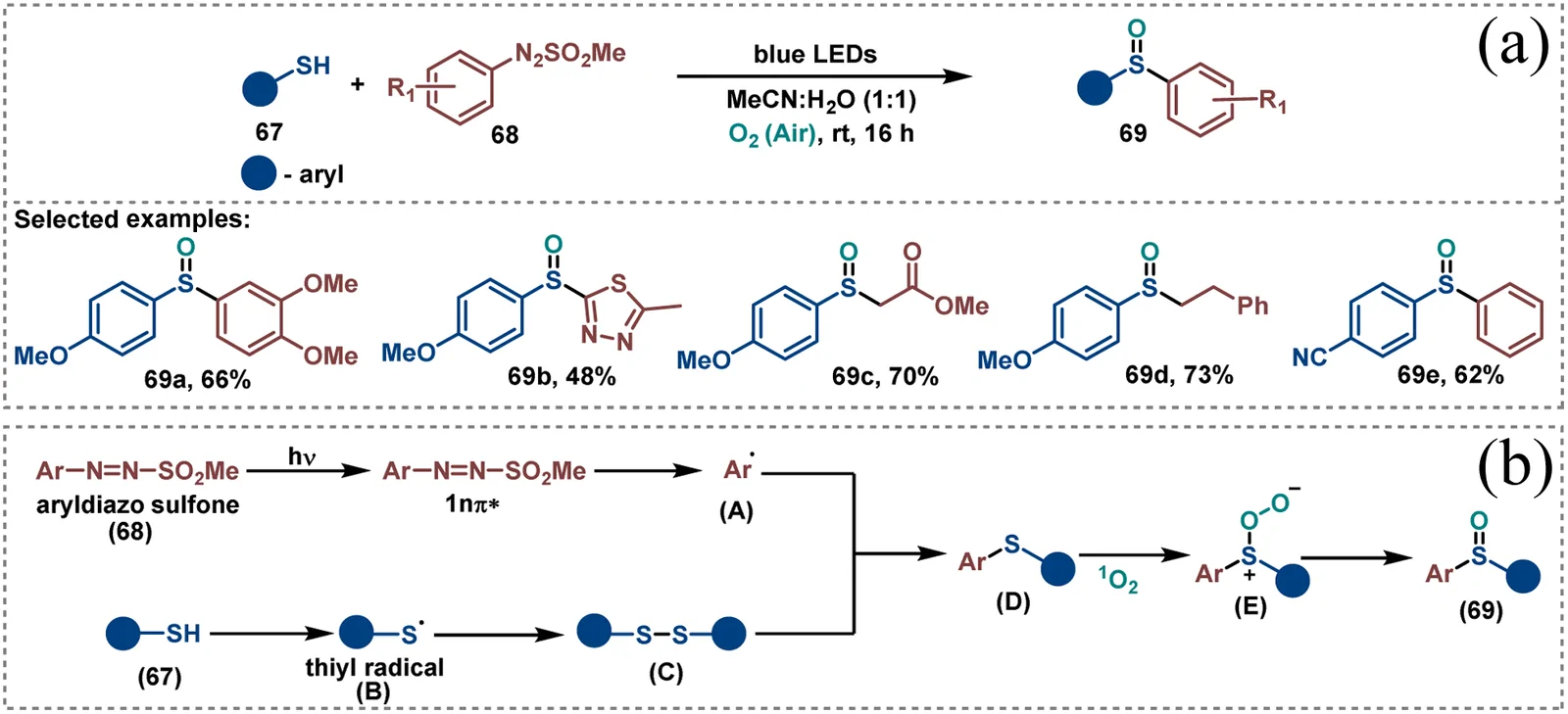
(a) Oxidative coupling of aryldiazo sulfones with thiols. (b) Proposed mechanism for oxidative coupling of aryldiazo sulfones with thiols.

Mechanistic investigations support a radical pathway in which photoexcited aryldiazo sulfones undergo homolytic N–S bond cleavage to generate aryl radicals (A), while thiols are oxidized by O_2_ to thiyl radicals that dimerize into disulfides (C) (Scheme 22b). The aryl radicals then couple with these disulfides to form sulfide intermediates (D), which are subsequently oxidized by singlet oxygen to the final sulfoxides. Radical-trapping, oxygen-dependence, and isotope-labeling experiments substantiate this radical-oxidation cascade. Overall, the method provides a mild, green, and operationally simple approach to structurally diverse sulfoxides using only visible light and atmospheric oxygen.

Hwang and co-workers in 2021 developed a visible-light-driven copper-photoredox protocol for the oxy-sulfonylation of terminal alkynes, enabling direct C–S bond formation to furnish β-keto sulfones under mild and environmentally benign conditions (Scheme 23a).^108^ The reaction employs CuI as the catalyst, 2-picolinic acid as the ligand, methanol as the solvent, TMSN_3_ as a key promoter, and blue LED irradiation under an atmospheric oxygen. This protocol uses bench-stable sodium sulfinates and simple terminal alkynes, avoids precious metals or strong oxidants, and provides β-keto sulfones in excellent yields with broad functional-group compatibility. A wide range of sodium sulfinates-bearing electron-donating, halogen, and strongly electron-withdrawing substituents (*e.g.*, –CF_3_, –OCF_3_, –CN, –NO_2_) reacted smoothly with phenylacetylene to afford β-keto sulfones in good to excellent yields. Heteroaryl sulfinates, including thiophene and naphthalene derivatives, were also well tolerated. Terminal alkynes bearing electron-rich (*e.g.*, –Me, –^*t*^Bu, –OMe, –OH), electron-poor (*e.g.*, –F, –Cl, –Br, –CF_3_, –CN, –NO_2_, –Ac, –COOMe), and halogen substituents delivered the corresponding β-keto sulfones with high efficiency. Bulky aryl alkynes and heteroaryl alkynes such as pyridyl and thiophenyl derivatives also participated effectively. The reaction displayed excellent chemoselectivity for terminal alkynes, with no formation of Glaser homocoupling products or over-oxidized byproducts. Aliphatic alkynes, however, were unreactive.

**Scheme 23:**
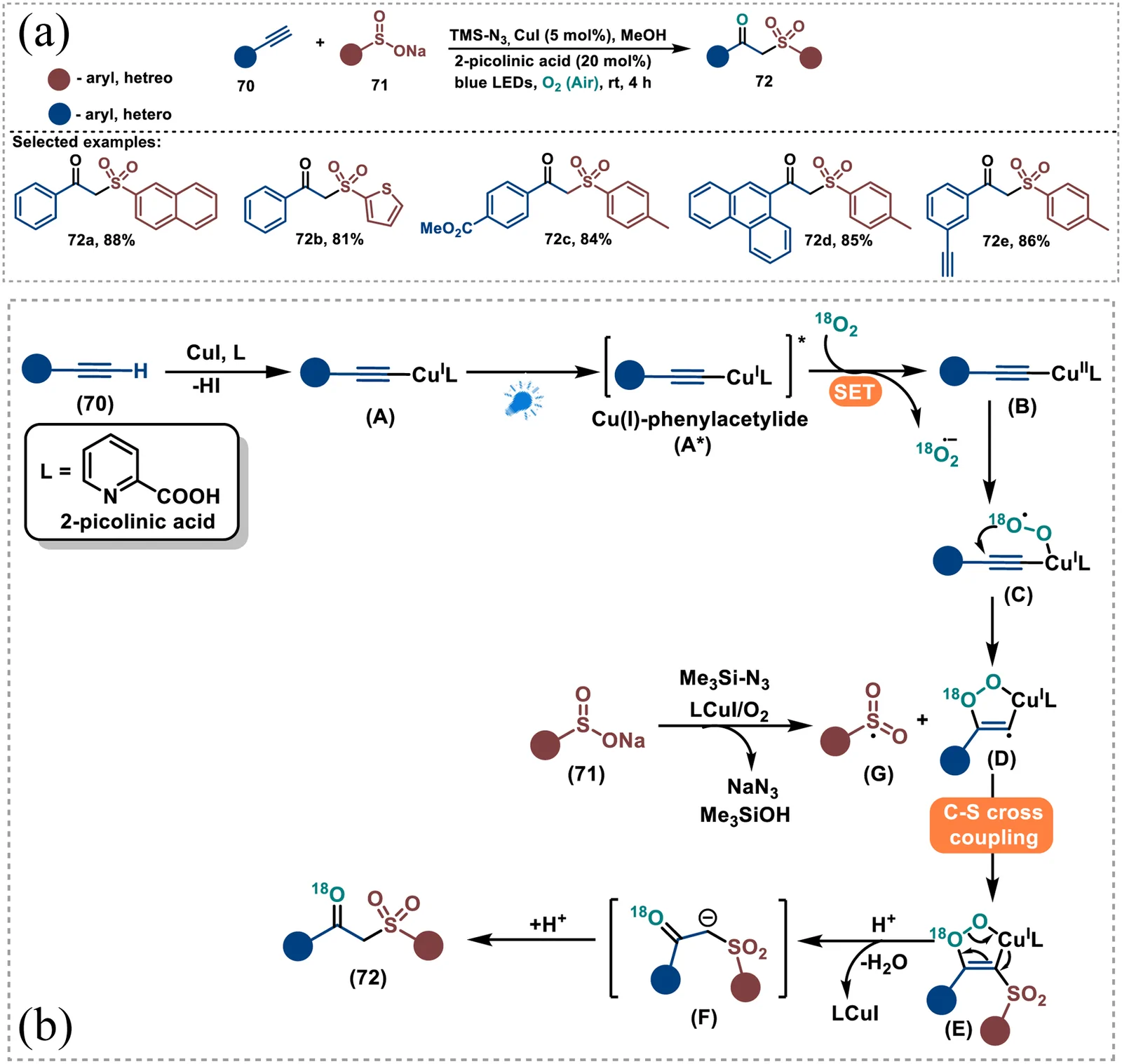
(a) Oxy-sulfonylation of terminal alkynes. (b) Proposed mechanism for oxy-sulfonylation of terminal alkynes.

Mechanistic studies support a copper-mediated photoredox pathway initiated by the *in situ* generation of a Cu(i)-phenylacetylide complex (A), which acts as the key light-absorbing species (Scheme 23b). Upon blue-light excitation, this complex reaches a long-lived triplet state and undergoes single-electron transfer (SET) to molecular oxygen, forming a superoxide radical anion and a Cu(ii)-acetylide (B). The resulting Cu(ii) species, together with superoxide, forms a Cu(i)-superoxo complex (C) capable of intramolecular radical attack on the C≡C bond, generating a cyclic radical intermediate (D). In parallel, sodium sulfinates react with TMSN_3_ under copper catalysis and O_2_ to produce sulfonyl radicals (G). Radical–radical coupling between the cyclic copper-bound radical intermediate and the sulfonyl radical affords a C–S coupled intermediate (E), which undergoes O–O bond cleavage, isomerization, and proton transfer to yield the β-keto sulfone. TEMPO inhibition and the requirement of O_2_ confirm the involvement of radical species, and ^18^O_2_-labeling studies demonstrate that the oxygen incorporated into the ketone originates from molecular oxygen. This method represents a green and highly efficient approach to β-keto sulfones, outperforming earlier thermal protocols in E-factor and overall sustainability metrics. Its operational simplicity, excellent atom economy, and broad synthetic utility-highlighted by access to biologically active molecules such as anti-analgesics, 11β-HSD1 inhibitors, and CES1 inhibitors-underscore the value of copper photoredox catalysis in modern C–S bond construction.

Mamat and co-workers in 2021 reported a base-promoted, visible-light-induced cascade reaction between epoxides and thiols, enabling the efficient synthesis of structurally important β-hydroxylsulfoxides under mild and sustainable conditions (Scheme 24a).^109^ The transformation utilizes rose bengal as an organic photocatalyst, ^i^Pr_2_NLi as a base, ethanol as the solvent, and molecular oxygen as the sole oxidant, with blue LED irradiation at room temperature. This protocol offers broad functional group tolerance and delivers products in moderate to excellent yields without requiring transition metals or stoichiometric oxidants. A variety of thiophenols, including those bearing electron-donating (*e.g.*, –Me, –^*t*^Bu, –OMe) and electron-withdrawing (*e.g.*, –F, –Cl, –Br) groups, were efficiently transformed into β-hydroxylsulfoxides (34–88% yields). Electron-deficient thiophenols generally gave higher yields, indicating the importance of thiol deprotonation. Steric hindrance had little effect, and even *ortho*-substituted thiophenols reacted well. The reaction also tolerated aliphatic thiols and naphthol derivatives. Epoxides bearing diverse functionalities such as trifluoromethyl, alkyl chloride, ester, hydroxyl, ether, alkyne, amine, and alkene groups participated smoothly, affording the desired products in 43–70% yields. Both monosubstituted and 2,3-disubstituted oxiranes were compatible with the reaction conditions.

**Scheme 24:**
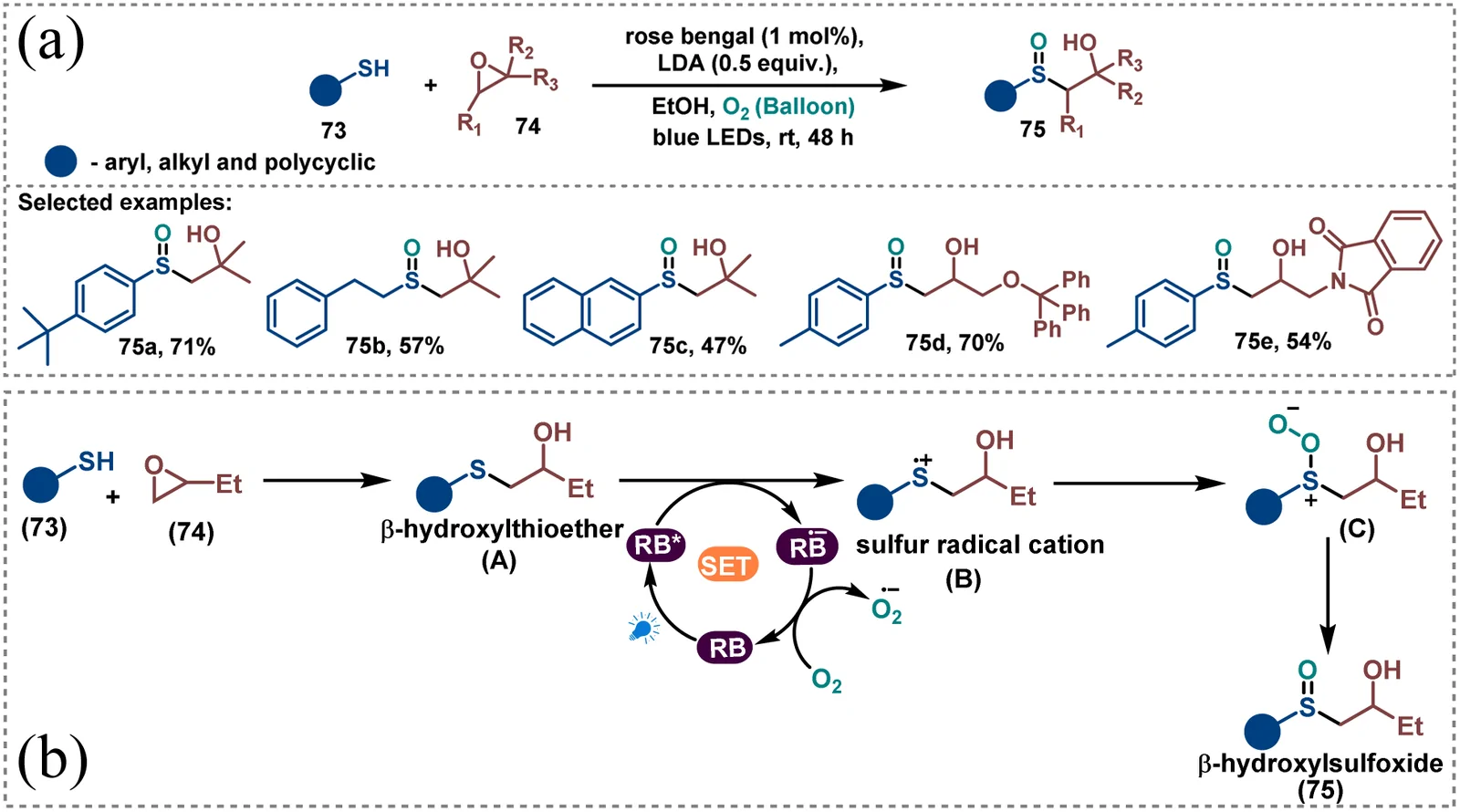
(a) Aerobic oxidation cascade reaction of epoxides and thiols. (b) Proposed mechanism for aerobic oxidation cascade reaction of epoxides and thiols.

Mechanistic studies suggest a two-step cascade sequence: base-promoted thiol addition to the epoxide, followed by visible-light-mediated aerobic oxidation (Scheme 24b). The first step proceeds *via* S_N_2 attack of the thiolate on the epoxide to yield β-hydroxylthioether (A). Under blue light irradiation, rose bengal in its excited state oxidizes this intermediate to a sulfur-centered radical cation (B), which couples with a peroxy radical (generated from oxygen reduction by the rose bengal radical anion) to form a peroxy intermediate. This intermediate subsequently reacts with another β-hydroxylthioether molecule to deliver the β-hydroxylsulfoxide. Control experiments confirmed the necessity of both light and photocatalyst for oxidation, and TEMPO trapping indicated radical involvement, while singlet oxygen was ruled out as an oxidant. This strategy provides a green, metal-free, and operationally simple route to β-hydroxylsulfoxides, highlighting the potential of combining organophotocatalysis with aerobic oxidation for efficient sulfur functionalization.

Chen and co-workers in 2022 reported a photocatalyst-free, visible-light-induced hydroxysulfenylation of styrenes using heterocyclic thiols under ambient air (Scheme 25a).^110^ The reaction proceeds in ethanol at room temperature and relies on the formation of an electron donor–acceptor (EDA) complex between the thiolate anion and styrene, eliminating the need for external photocatalysts, metals, or peroxides. A variety of heterocyclic thiols, including benzimidazole-, benzothiazole-, benzoxazole-, and thiadiazole-based thiols, reacted efficiently to form β-hydroxysulfides in good to excellent yields. Diverse styrenes, especially 1,1-diphenylethylenes bearing electron-donating or electron-withdrawing groups, also showed excellent reactivity. Heteroaryl alkenes such as thiophene- and naphthalene-substituted alkenes participated smoothly. Simple styrenes and thiophenols gave lower yields, while aliphatic thiols and unactivated alkenes were unreactive due to their inability to form EDA complexes.

**Scheme 25:**
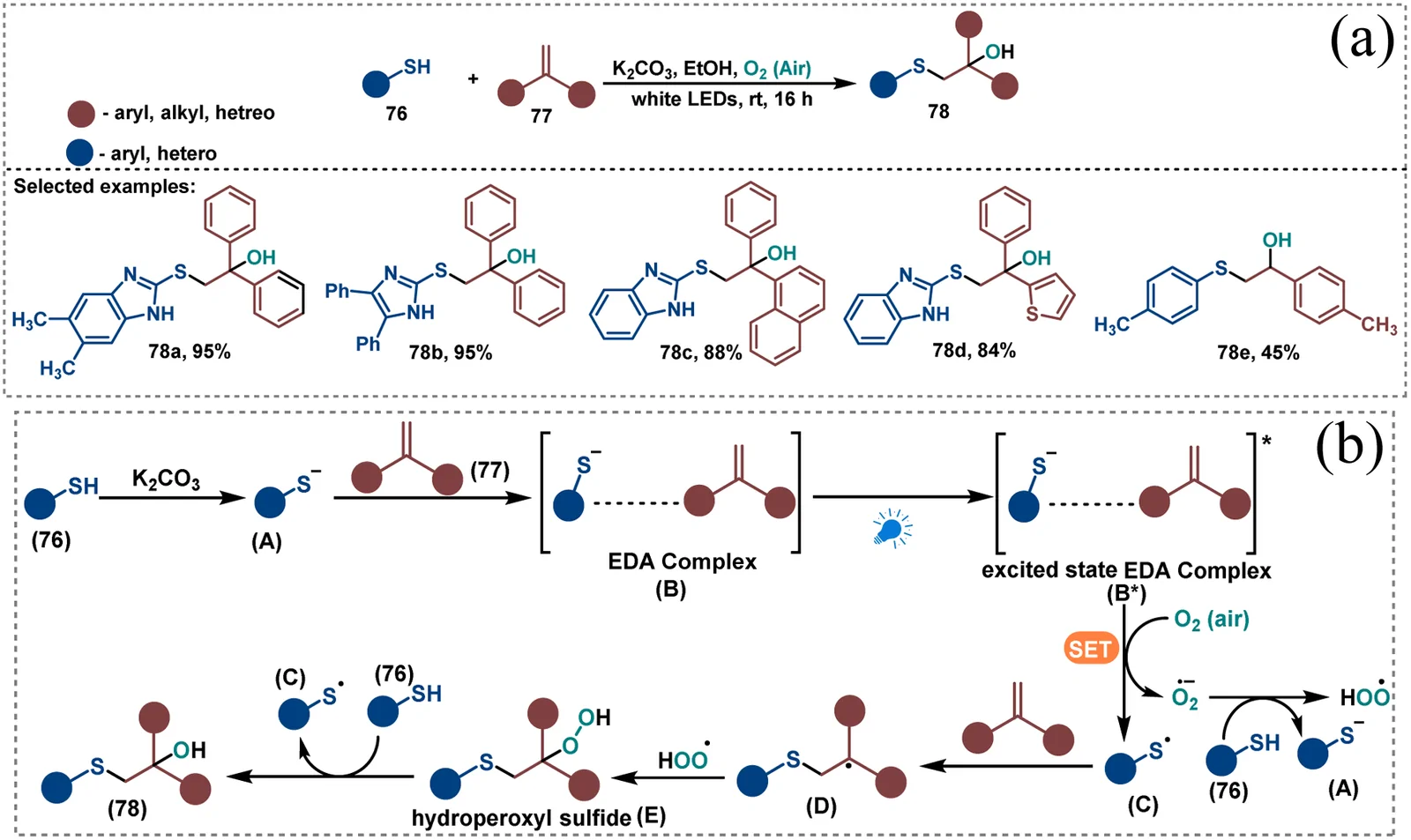
(a) EDA complex-promoted hydroxysulfenylation of styrenes. (b) Proposed mechanism for EDA complex-promoted hydroxysulfenylation of styrenes.

Mechanistic studies support a visible-light-induced EDA-complex radical pathway (Scheme 25b). Deprotonation of the heterocyclic thiol by K_2_CO_3_ generates a thiolate anion (A), which forms an EDA complex with the styrene. Upon visible-light excitation, this complex undergoes single-electron transfer to produce a sulfur-centered radical and superoxide (O_2_˙^−^). The sulfur radical adds to the alkene to form a benzylic radical (D), which couples with a peroxide radical derived from superoxide to generate a peroxy-sulfide intermediate (E). Cleavage of the O–O bond, assisted by another molecule of thiol, affords the β-hydroxysulfide product. Radical trapping (TEMPO, BHT) suppressed reactivity, and benzoquinone inhibition confirmed (O_2_˙^−^) involvement. ^18^O_2_-labeling established that the hydroxyl oxygen originates from molecular oxygen rather than solvent water. UV-vis and NMR studies further verified *in situ* EDA complex formation. This EDA-enabled, photocatalyst-free strategy represents a green and efficient platform for β-hydroxysulfide synthesis, offering high atom economy, broad functional-group tolerance, and practical scalability-even under natural sunlight.

Zhou, Li, and Sun in 2023 developed a green and efficient photocatalytic protocol for synthesizing α-keton thiol esters through the oxidative radical addition of thioic acids to alkenes under visible light (Scheme 26a).^111^ The method uses thioxanthone as an inexpensive organic photocatalyst, EtOAc as a green solvent, and molecular oxygen as the sole oxidant, producing only water as the byproduct. A broad variety of alkenes-including styrene, substituted styrenes, and 1,1-disubstituted alkenes-reacted smoothly to furnish α-keton thiol esters in moderate to excellent yields. Electron-rich groups such as (*e.g.*, –Me, –^*t*^Bu, –OMe, and amines) were well tolerated, while electron-withdrawing substituents like (*e.g.*, –F, –Cl, –Br, –CF_3_, –NO_2_, and –CO_2_Me) also provided good yields with slight decreases in strongly withdrawing cases. Both aromatic and aliphatic alkenes were compatible. A wide range of thioic acids, including electron-rich, electron-poor, and aliphatic variants, also participated effectively. The method was successfully applied to late-stage functionalization of natural-product-based alkenes, demonstrating synthetic utility.

**Scheme 26:**
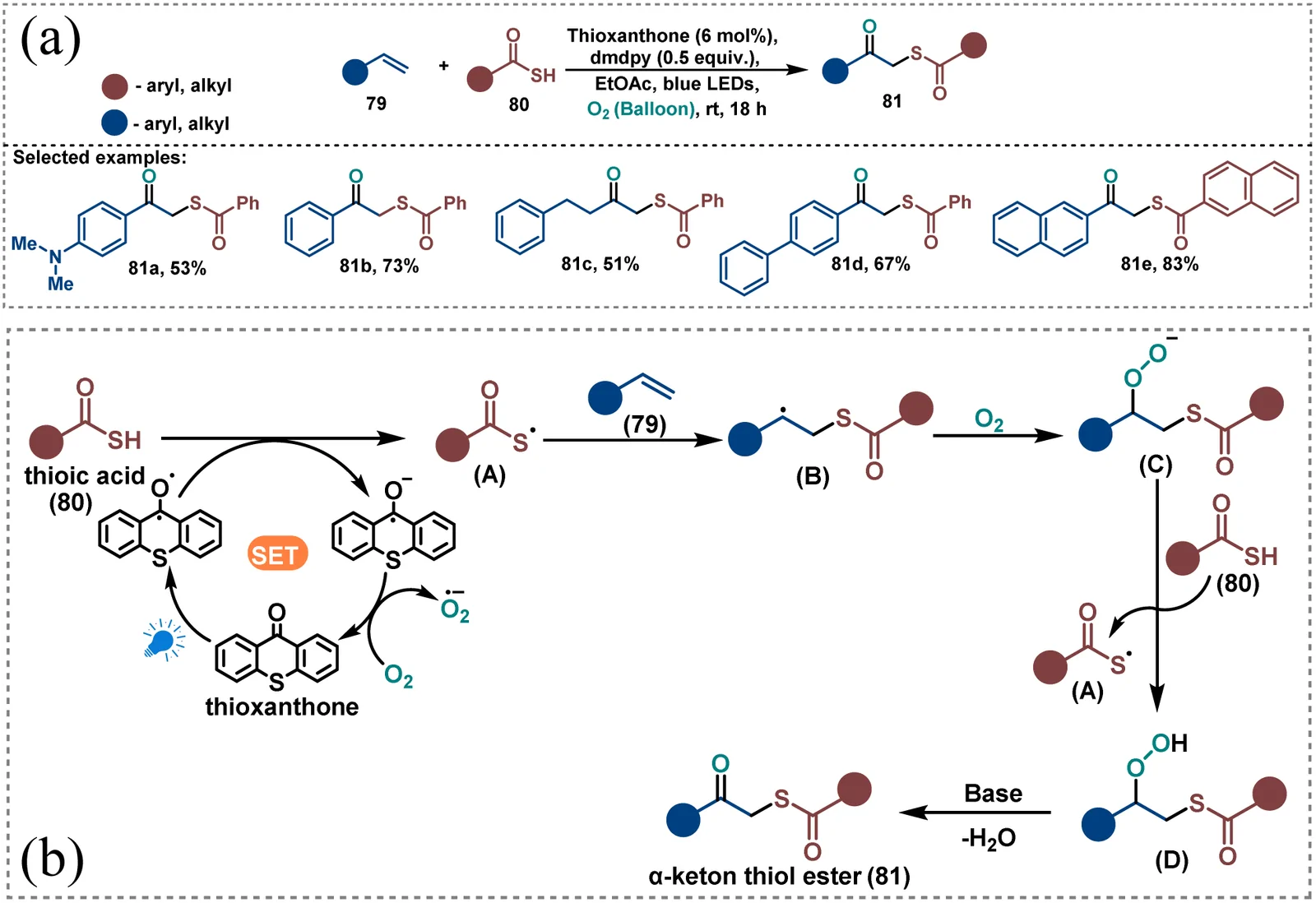
(a) Oxidative radical addition of thioic acid to alkene. (b) Proposed mechanism for oxidative radical addition of thioic acid to alkene.

Mechanistic studies support a radical pathway requiring light, photocatalyst, base, and oxygen. Stern–Volmer quenching confirmed that thioic acid quenches the excited photocatalyst (Scheme 26b). Under blue LED irradiation, thioxanthone oxidizes the thioic acid to generate a thioacyl radical (A), which adds to the alkene to form intermediate (B). This carbon-centered radical is trapped by molecular oxygen to produce peroxy intermediate (C), which undergoes proton abstraction and subsequent dehydration to furnish the α-keton thiol ester. The reaction is fully suppressed by TEMPO, confirming the involvement of radical intermediates. Control studies also showed that the transformation does not occur without light, oxygen, base, or photocatalyst. Overall, this photocatalytic oxidative radical addition provides a mild, sustainable, and operationally simple method for constructing α-keton thiol esters, aligning strongly with green-chemistry principles and significantly expanding the synthetic toolbox for α-keton sulfide derivatives.

By combining (3-chloroprop-1-yn-1-yl)benzene with thiols under moderate circumstances, Rizvi and colleagues in 2026 created a one-step photoredox-mediated process for the production of propargylic sulfoxides (Scheme 27a).^112^ The technique uses methanol as the solvent, triethylamine as a base, and Ru(bpy)_3_Cl_2_ as a photocatalyst under blue LED irradiation. With oxygen from the air serving as the oxidant, the protocol notably removes the requirement for external oxidants, providing a simplified and sustainable method for sulfoxide synthesis.

**Scheme 27:**
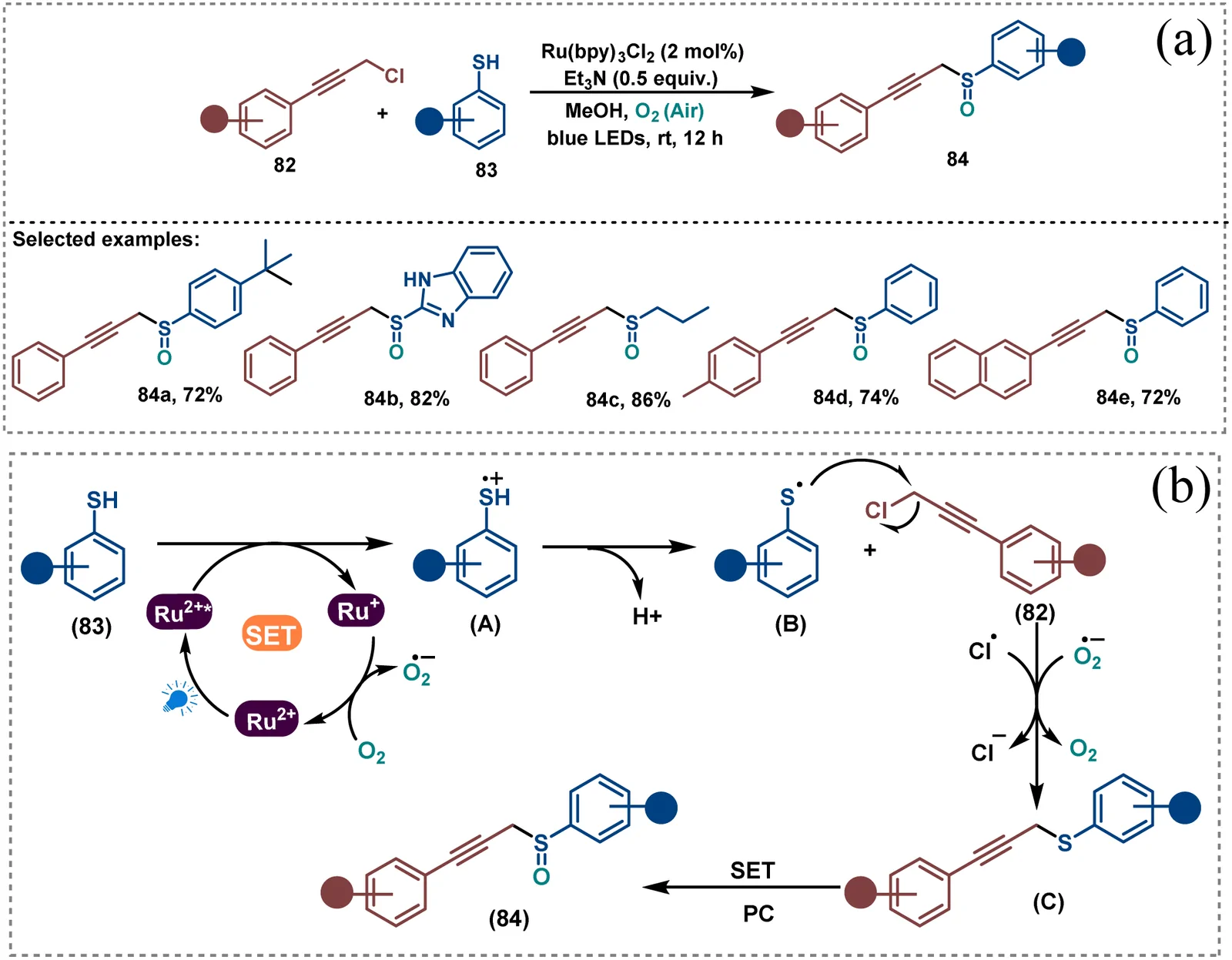
(a) Photoredox-mediated process for the production of propargylic sulfoxides. (b) Proposed mechanism for photoredox-mediated process for the production of propargylic sulfoxides.

The required propargylic sulfoxides were produced in good to outstanding yields (65–81%) from a variety of thiols, including electron-rich, electron-deficient, and halogen-substituted aryl thiols. The method's wide application was demonstrated by the effective participation of heterocyclic and aliphatic thiols. Similar to this, a variety of alkynes with heteroaryl, electron-donating, and electron-withdrawing substituents were compatible and produced the appropriate compounds in moderate to good yields. Nevertheless, several heterocyclic thiols produced sulfides rather than sulfoxides, suggesting restrictions on selectivity.

A radical pathway involving thiyl radical intermediates was verified by mechanistic investigations, such as radical entrapment and Stern–Volmer experiments (Scheme 27b). The excited photocatalyst experiences reductive quenching when exposed to light, producing thiyl radicals (B) that combine with the alkyne substrate to create propargyl sulfide intermediates (C). The required sulfoxide compounds are formed through further oxidation by molecular oxygen. Continuous light irradiation and oxygen are necessary for the transformation, as further demonstrated by control studies. Although issues like overoxidation control and substrate-dependent selectivity still exist, this technique generally offers a direct, effective, and atom-economical pathway to propargylic sulfoxides with good chemo-selectivity.

Sun and colleagues in 2021 devised a chemoselectivity-controlled, visible light-driven method for functionalizing 2*H*-indazoles through the creation of C–O bonds with alcohols (Scheme 28a).^113^ The technique uses Mes-Acr^+^ClO_4_^−^ as an organic photocatalyst under blue LED irradiation, where the environment (N_2_*versus* O_2_) controls the reaction pathway. C3-alkoxylated 2*H*-indazoles are produced in large quantities using a cross-dehydrogenative coupling process in a nitrogen atmosphere with Selectfluor. On the other hand, the reaction continues by ring-opening to produce unsymmetrical *ortho*-alkoxycarbonylated azobenzenes in an oxygen atmosphere without Selectfluor.

**Scheme 28:**
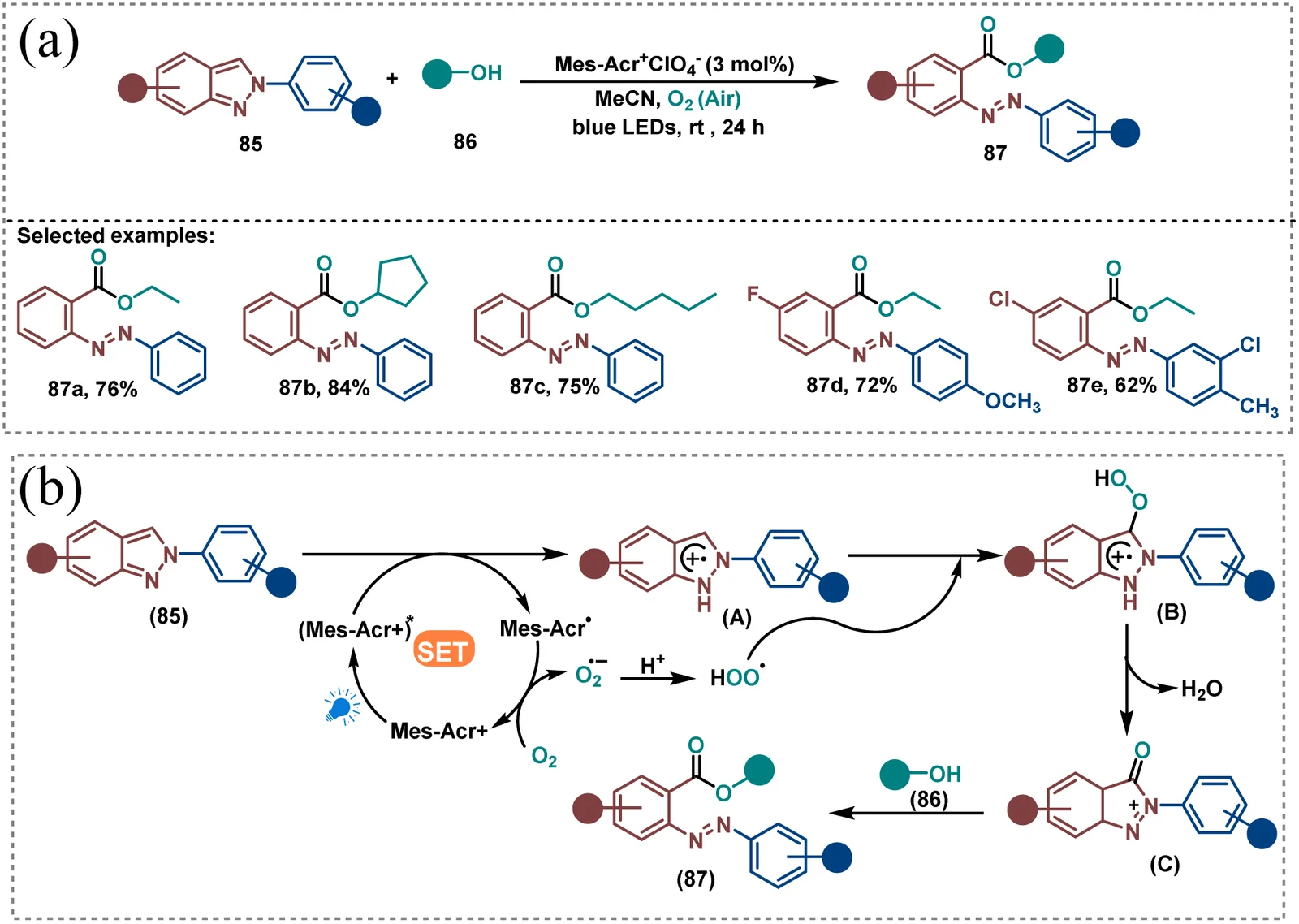
(a) Visible light-driven method for functionalizing 2*H*-indazoles through the creation of C–O bonds with alcohols. (b) Proposed mechanism for the visible light-driven method for functionalizing 2*H*-indazoles through the creation of C–O bonds with alcohols.

Along with a variety of 2-aryl-2*H*-indazoles with electron-donating and electron-withdrawing substituents that exhibited little electronic bias, a wide range of alcohols, including primary, secondary, and cyclic systems, were well tolerated. However, other alcohols, such as phenols, and sterically hindered substrates were not reactive.

A radical route started by photoinduced single-electron transfer is supported by mechanistic investigations; in an oxygen atmosphere, superoxide radicals are crucial, and in a nitrogen atmosphere, Selectfluor facilitates the transfer of hydrogen atoms (Scheme 28b). Light, photocatalyst, and environment are crucial for regulating the reaction's result, according to control tests. Sensitivity to steric effects and dependence on particular reaction conditions are still drawbacks, but overall, this process offers a flexible and diverse strategy for C–O bond production.

A novel class of organic photocatalysts based on triaryl-heptazine for oxidative transformations of nitrogen-containing compounds was created by Montinho-Inacio and colleagues in 2026 (Scheme 29a).^114^ These catalysts were created to overcome the drawbacks of traditional organic photocatalysts, such as short lives or inadequate redox power, by combining high excited-state oxidation potentials (up to +1.69 V *versus* SCE) with comparatively long excited-state durations (23–72 ns). A flexible platform for photocatalyst design is made possible by the modular triaryl substitution, which allows fine-tuning of photophysical and electrical properties.

**Scheme 29:**
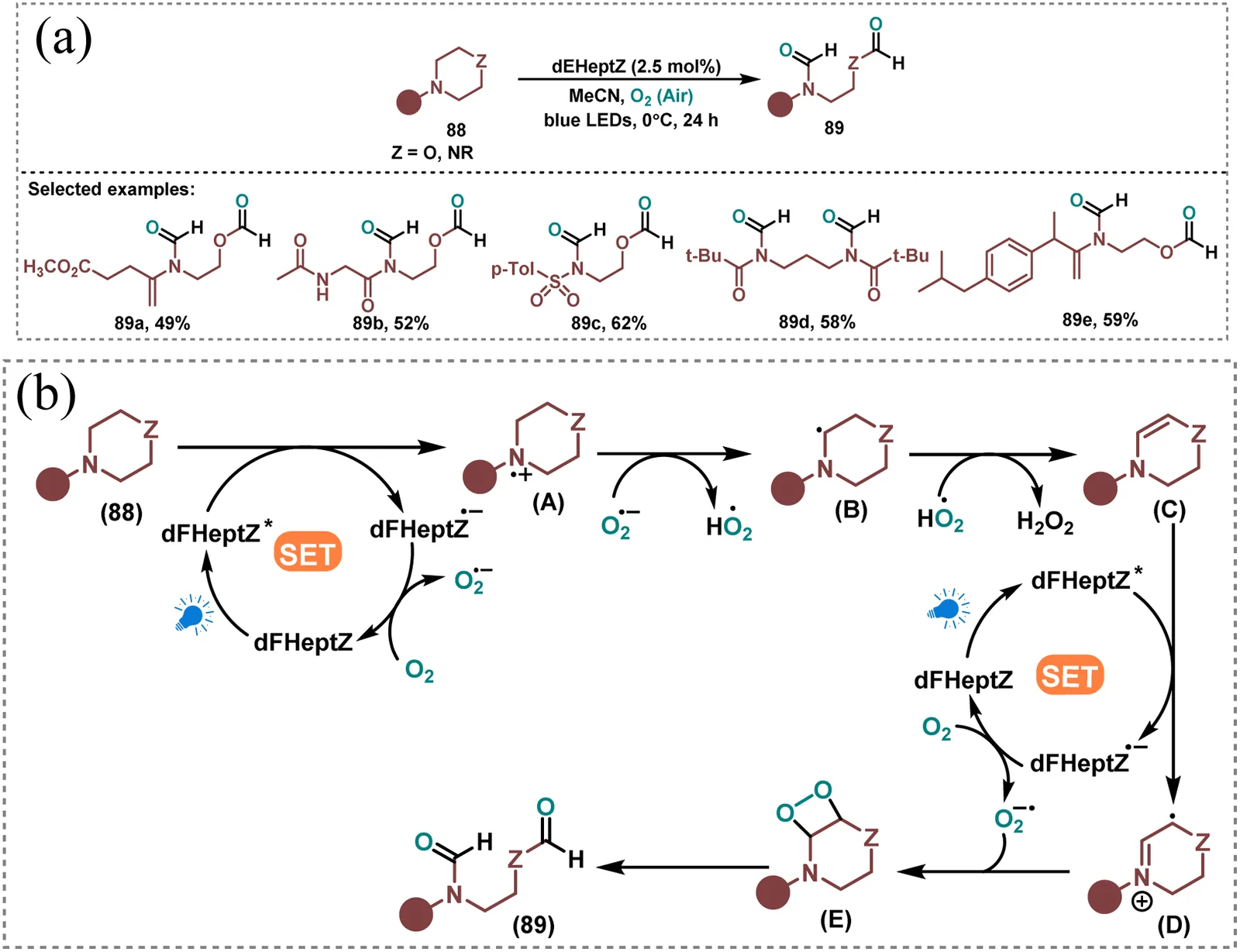
(a) Triaryl-heptazine for oxidative transformations of nitrogen-containing compounds. (b) Proposed mechanism of triaryl-heptazine for oxidative transformations of nitrogen-containing compounds.

The improved heptazine catalyst effectively encourages the selective oxidation of *N*-methyl groups in protected amines under ambient air conditions and visible light irradiation, producing *N*-formyl derivatives in moderate to good yields. Broad functional group tolerance was demonstrated by the effective transformation of a variety of substrates, including amides, carbamates, ureas, and sulfonamides. Notably, even in structurally complex compounds with numerous possible reactive sites, the approach demonstrates significant site-selectivity for *N*-methyl oxidation. The catalyst's synthetic value is further demonstrated by the fact that it permits oxidative C–C bond cleavage in nitrogen-containing heterocycles in addition to C–H oxidation.

A photoinduced electron-transfer pathway powered by the highly oxidizing excited state of the heptazine catalyst is suggested by mechanistic investigations, which are corroborated by photophysical experiments and DFT calculations (Scheme 29b). Atmospheric oxygen acts as the terminal oxidant in mild circumstances, and the lengthy excited-state lifespan promotes effective substrate interaction. The critical functions of light and oxygen in the transformation are confirmed by control studies. All things considered, this work presents a potent and adjustable organic photocatalyst platform for difficult oxidative reactions. However, there are also drawbacks, such as reliance on an oxygen atmosphere and limited yields in some situations. However, this work offers a promising method for creating next-generation high-potential organic photocatalysts and greatly broadens the application of heptazine-based photocatalysis.

## 3 Summary and outlook

Visible-light photoredox catalysis has revolutionized the activation and functionalization of inert C–H bonds by offering mild, green, and atom-efficient pathways for constructing C–C and C–heteroatom bonds. The use of molecular oxygen as both a terminal oxidant and an oxygen-atom donor has significantly expanded the scope of oxygenative coupling reactions, enabling a wide array of transformations ranging from aerobic oxidation of alkynes and annulative synthesis of quinazolines to oxidative cleavage, hydroacylation, and metal-free radical-mediated functionalizations. Mechanistic studies across diverse systems highlight the pivotal roles of reactive oxygen species, radical intermediates, energy-transfer pathways, and the formation of dioxetane/oxetane intermediates. Collectively, recent advancements establish oxygen-enabled photoredox catalysis as an indispensable tool in modern synthetic chemistry, offering sustainable alternatives to traditional oxidation-based methodologies.

Despite remarkable progress, substantial opportunities exist for further innovation in oxygenative C–H functionalization under photoredox conditions. Future developments may focus on expanding substrate generality to unactivated aliphatic systems, designing photocatalysts with higher quantum efficiency, and achieving selective activation in complex molecular settings. The emergence of catalyst-free and EDA-complex-driven strategies signals a promising direction toward simpler, more economical systems. Additionally, integrating photoredox catalysis with flow chemistry, electrochemistry, or enzymatic platforms could unlock unprecedented levels of efficiency and selectivity. Mechanistic elucidation particularly regarding oxygen activation pathways and short-lived radical intermediates will continue to guide reaction design. With increasing emphasis on sustainability, the union of visible-light catalysis and molecular oxygen is poised to play a central role in the development of greener synthetic technologies and late-stage functionalization strategies in pharmaceuticals, materials science, and fine-chemical synthesis.

## Conflicts of interest

There are no conflicts to declare.

## Data Availability

No primary research results, software or code have been included, and no new data were generated or analysed as part of this review.
